# Mitochondria dysfunction impairs *Tribolium castaneum* wing development during metamorphosis

**DOI:** 10.1038/s42003-022-04185-z

**Published:** 2022-11-15

**Authors:** Yaoyu Jiao, Subba Reddy Palli

**Affiliations:** Department of Entomology, College of Agriculture, Food and Environment, University of Kentucky, Lexington, KY 40546 USA

**Keywords:** Entomology, Animal physiology

## Abstract

The disproportionate growth of insect appendages such as facultative growth of wings and exaggeration of beetle horns are examples of phenotypic plasticity. Insect metamorphosis is the critical stage for development of pupal and adult structures and degeneration of the larval cells. How the disproportionate growth of external appendages is regulated during tissue remodeling remains unanswered. *Tribolium castaneum* is used as a model to study the function of mitochondria in metamorphosis. Mitochondrial dysfunction is achieved by the knockdown of key mitochondrial regulators. Here we show that mitochondrial function is not required for metamorphosis except that severe mitochondrial dysfunction blocks ecdysis. Surprisingly, various abnormal wing growth, including short and wingless phenotypes, are induced after knocking down mitochondrial regulators. Mitochondrial activity is regulated by IIS (insulin/insulin-like growth factor signaling)/FOXO (forkhead box, sub-group O) pathway through TFAM (transcription factor A, mitochondrial). RNA sequencing and differential gene expression analysis show that wing-patterning and insect hormone response genes are downregulated, while programmed cell death and immune response genes are upregulated in insect wing discs with mitochondrial dysfunction. These studies reveal that mitochondria play critical roles in regulating insect wing growth by targeting wing development during metamorphosis, thus showing a novel molecular mechanism underlying developmental plasticity.

## Abstract (web summary)

Mitochondrial disruption in the red flour beetle leads to insects with three layers of cuticles (larval, pupal, and adult) and blocks the wing development.

## Introduction

Insect metamorphosis is a fascinating developmental process in which the insect body is transformed from juvenile to mature adult, morphologically and physiologically distinct from juvenile. This provides a valuable opportunity to study the fundamentals of development and evolution. In hemimetabolous insects, adult structures such as wings and reproductive system rapidly develop during the last molt from nymph to adult. In contrast, in holometabolous insects, most adult organs, including digestive and nervous systems and appendages such as wings, antennae, and genitals, begin to develop just before entering the pupal stage. Developmental plasticity of adult structures was found in insects from both types of metamorphosis represented by disproportionate adult appendages. For example, some beetles grow enlarged horns or mandibles when experiencing a superior nutritional condition, which helps to increase intraspecific competitiveness^1,2^. On the contrary, some insects diminish the growth of specific appendages. The growth of wings can be selectively inhibited or retained in response to environmental signaling to balance the trade-off between reproduction and dispersal. This phenomenon was called wing polyphenism^3^. Wing polyphenism is widely observed in many insects belonging to orders Hemiptera, Coleoptera, Hymenoptera, Orthoptera, Diptera, Lepidoptera, Isoptera, Psocoptera, and Dermaptera^4^. Since the disproportionate growth of these adult appendages mainly occurs during insect metamorphosis, the regulation during metamorphosis could be essential to the developmental plasticity of insect adult appendages.

Nutrition is one of the most widespread environmental factors that induce phenotypic plasticity during animal development. Previous studies showed that IIS (insulin/insulin-like growth factor signaling)/FOXO (forkhead box, sub-group O) is the key signaling pathway regulating nutrition-responsive developmental plasticity, including both outgrowth of insect weapons and diminishment of insect wings^1,5–7^. The IIS/FOXO is an evolutionarily conserved signaling pathway from insects to humans that regulates varied biological processes, including development, growth, metabolism, and reproduction. IIS pathway has been widely reported to regulate organism growth and affect final body size^8^. Suppression of IIS pathway causes a delayed developmental rate and reduction in the whole body and organ size in *Drosophila melanogaster*^9^. FOXO is one of the main negative regulators in IIS pathway. Overexpression of *FOXO* in *D. melanogaster* induced a decrease in cell number and growth and caused a reduction in body size^10,11^. Knockdown of *FOXO* does not affect *D. melanogaster* body size but reduces food intake and body size of *T. castaneum*^11,12^.

For the downstream regulation of IIS signaling, two mechanisms involved in regulating insect allometric growth of disproportionate appendages have been described previously. The first one is organ-specific manipulation of IIS/FOXO signaling. The male genitalia of *D. melanogaster* is known to be less nutrition-sensitive compared to other tissues such as wings. Tang et al. showed that maintaining low expression levels of *FOXO* gene under a low-nutrition environment is critical for *D. melanogaster* male genitalia to sustain a constant size^13^. In contrast, beetle horn with a high sensitivity to nutrition has a strikingly high *FOXO* expression compared to that in genitalia or brain^6,14^. Specific tissues may also hijack the IIS signaling by producing other regulators. Cheng et al. revealed that *D. melanogaster* central nervous system growth is protected against starvation by releasing the Jelly belly protein to directly activate the IIS signaling bypassing insulin receptors^15^. Secondly, IIS/FOXO signaling was reported to regulate transcription factors of specific tissues or sex to achieve tissue-specific growth. In the brown planthopper (*Nilaparvata lugens*), FOXO was reported to temporally regulate the expression of *vestigial* gene, which encodes a key nuclear protein regulating wing formation^16^. Recent studies showed that doublesex, a cardinal member of sex determination, and Hedgehog signaling is critical for regulating the nutrition-sensitive growth of male beetle horns, and this may be regulated by IIS/FOXO signaling^6,14^.

Mitochondria play an irreplaceable role in converting nutrition to energy through oxidative phosphorylation. In addition to serving as the powerhouse of all animal cells, mitochondrion also functions to maintain calcium homeostasis, generation of reactive oxygen species (ROS), apoptosis, and metabolism^17^. Mitochondrial function is known to be regulated by insulin signaling cascade^18^. On the contrary, mitochondrial metabolism can also regulate insulin signaling to control body development^19^. It is not clear if mitochondria are involved in insect developmental plasticity, despite the evidence indicating the involvement of insulin signaling in regulating disproportionate appendage growth in several species^1,2,5,7,20–22^. A recent paper showed the first evidence of mitochondrial involvement in the wing polyphenism of the pea aphid *Acyrthosiphon pisum*, the flight-muscle degeneration during the dispersal to reproduction transition was induced by the mitochondrial dysfunction regulated by increased nutrition-dependent juvenile hormone (JH) sensitivity^23^. The mitochondrial structure and function rely on mitochondrial proteins that are encoded by nuclear genes. *Leucine-Rich Pentatricopeptide Repeat Containing* (*LRPPRC*) is one nuclear gene encoding an essential mitochondrial post-transcriptional regulator^24,25^. *LRPPRC* was first identified in Leigh syndrome French-Canadian, a rare type of severe human neurological disorder caused by its mutation. Two *LRPPRC* genes have been identified in *Drosophila melanogaster*, including *DmLRPPRC1* and *DmLRPPRC2*^26^. Like its mammalian counterpart, DmLRPPRC1 regulates mitochondrial mRNAs (mt-mRNA) polyadenylation and maturation, while DmLRPPRC2 coordinates translation by organizing the entrance of mt-mRNAs into ribosomes^26,27^. Only one homolog of *LRPPRC* was identified in the red flour beetle *T. castaneum*, the model insect we used in this study.

In the present study, we uncovered a potential mechanism of mitochondria regulating insect disproportionate appendage growth during metamorphosis. We induced the mitochondrial dysfunction in *T. castaneum* by knocking down *TcLRPPRC*. It was surprisingly found that shutting down mitochondrial genome transcription and/or translation through *TcLRPPRC* knockdown severely inhibits insect wing growth without affecting the remodeling of other insect tissues during metamorphosis. Different abnormal wings including wingless and short-wing phenotypes were observed from mitochondrial dysfunction to a varying extent by knocking down other mitochondrial regulators. We further show and discuss how mitochondria respond to IIS/FOXO signaling and regulate wing growth plasticity. These findings advance our understanding of the molecular mechanism of wing development and polyphenism.

## Results

### Knockdown of a mitochondrial regulator, *TcLRPPRC*, causes ecdysis failure without blocking metamorphosis

**Fig. 1:**
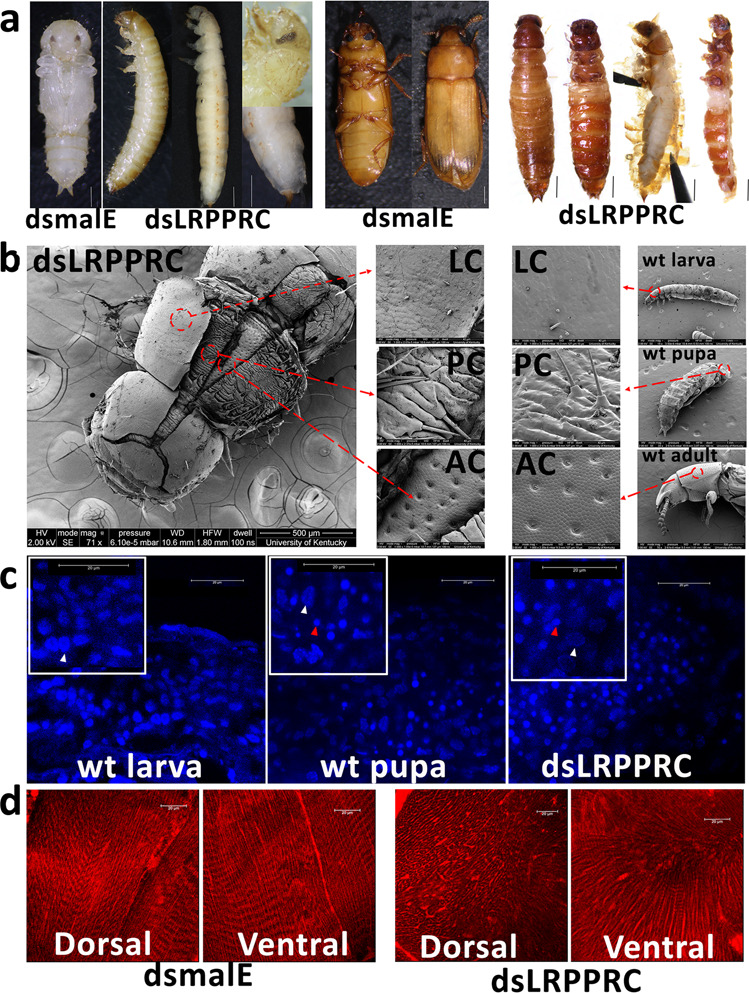
Knockdown of *TcLRPPRC* blocks *T. castaneum* ecdysis. **a** *dsmalE* and *dsTcLRPPRC* were injected in the newly molted last instar larvae. Control insects injected with *dsmalE* developed normally and pupated. Insects injected with *dsTcLRPPRC* failed to pupate; photographs of them before and after peeling off the larval cuticle are shown. Pictures of insects in the pupal stage were taken on the 6th day after injection. Control adults treated with *dsmalE* eclosed into adults. Insects treated with *dsTcLRPPRC* survived into the adult stage without ecdysis of both larval and pupal cuticles; photographs of them before and after peeling off old cuticles were shown. Pictures of insects in the adult stage were photographed on the 13th day after injection. Scale bar = 1 mm. **b** The SEM images of insects treated with *dsTcLRPPRC*. The outer cuticles were peeled off using forceps to see the inside of cuticle. Insects were sampled on the 13th day after injection. Wild-type larva, pupa, and adult were collected after their ecdysis to respective stages. Areas marked with red circles were enlarged in the two middle panels. LC, larval cuticle; PC, pupal cuticle; AC, adult cuticle. **c** DAPI-stained midguts of wild-type larva, newly molted pupa, and *dsTcLRPPRC* injected larvae on 6th day after dsRNA injection. White arrows indicate larval polyploid cells with large nuclei and red arrows mark intestinal stem cells with small nuclei. **d** Images of body muscle. Insects treated with *dsmalE* or *dsTcLRPPRC* were sampled at the prepupal stage. The first and second abdominal segments were cut, cleaned, stained with MitoView™ 650, and imaged under a confocal microscope. Scale bar = 20 μm.

To study the function of mitochondria in insect metamorphosis, we knocked down the single homolog of *LRPPRC* (*TcLRPPRC*) in *T. castaneum* by injection of dsRNA into newly molted last instar larvae. Larvae treated with *TcLRPPRC* dsRNA (*dsTcLRPPRC*) failed to undergo ecdysis into the pupal stage (Fig. 1a). Pupal/adult structures such as compound eyes, antenna, gin traps and genitals were found after peeling off old larval cuticle in these insects (Fig. 1a). When insects injected with control *malE* dsRNA (*dsmalE*) eclosed as adults, the *dsTcLRPPRC* injected insects failed to undergo adult ecdysis. However, sclerotized cuticle structures were found on the ventral abdomen, thorax, and head of these insects (Fig. 1a). To determine the identity of the sclerotized cuticle, a scanning electron microscope (SEM) was used to characterize cuticles. Three layers of cuticles were detected in *TcLRPPRC* knockdown insects (Fig. 1b). By comparison with the cuticles from wild-type insects, we determined that the three layers of cuticles are larval, pupal, and adult cuticles (Fig. 1b). During insect metamorphosis, several tissues, including the midgut, undergo remodeling; larval cells are eliminated through programmed cell death and the stem cells divide and differentiate to form pupal/adult tissues^28,29^. To verify whether midgut remodeling occurred in *dsTcLRPPRC* injected insects, the midguts dissected from wild-type and *dsTcLRPPRC* injected insects were compared. Microscopy observation of midguts exposed to nuclear stain DAPI showed no significant difference in the remodeling of midgut between control and *dsTcLRPPRC* injected insects (Fig. 1c and Supplementary Fig. 1). The alimentary canals dissected from the control larvae, pupae and adults showed remodeling of the larval alimentary canal to pupal/adult alimentary canal, while the alimentary canal dissected from the insects injected with *dsTcLRPPRC* is like that seen in control pupae and adults (Supplementary Fig. 1). The midguts from control larvae showed large polyploid nuclei and the midgut from the pupae, adults or *dsTcLRPPRC* injected insects showed smaller diploid nuclei (Fig. 1c). These data suggest that knockdown of *TcLRPPRC* blocks ecdysis, but the development of other tissues continues. To determine the reasons for ecdysis failure, confocal microscopy was used to study integument muscle development in insects treated with *dsTcLRPPRC* or *dsmalE*. Both dorsal and ventral muscles of *dsTcLRPPRC*-treated insects showed defects in development and organization when compared to those in control insects (Fig. 1d). In addition to the ecdysis failure, surprisingly, a wingless phenotype was found in these insects treated with *dsTcLRPPRC*. Both elytra and hind wings are absent in these insects (Fig. 1a).

### TcLRPPRC regulates both stability of mitochondrial transcripts and their translation

**Fig. 2:**
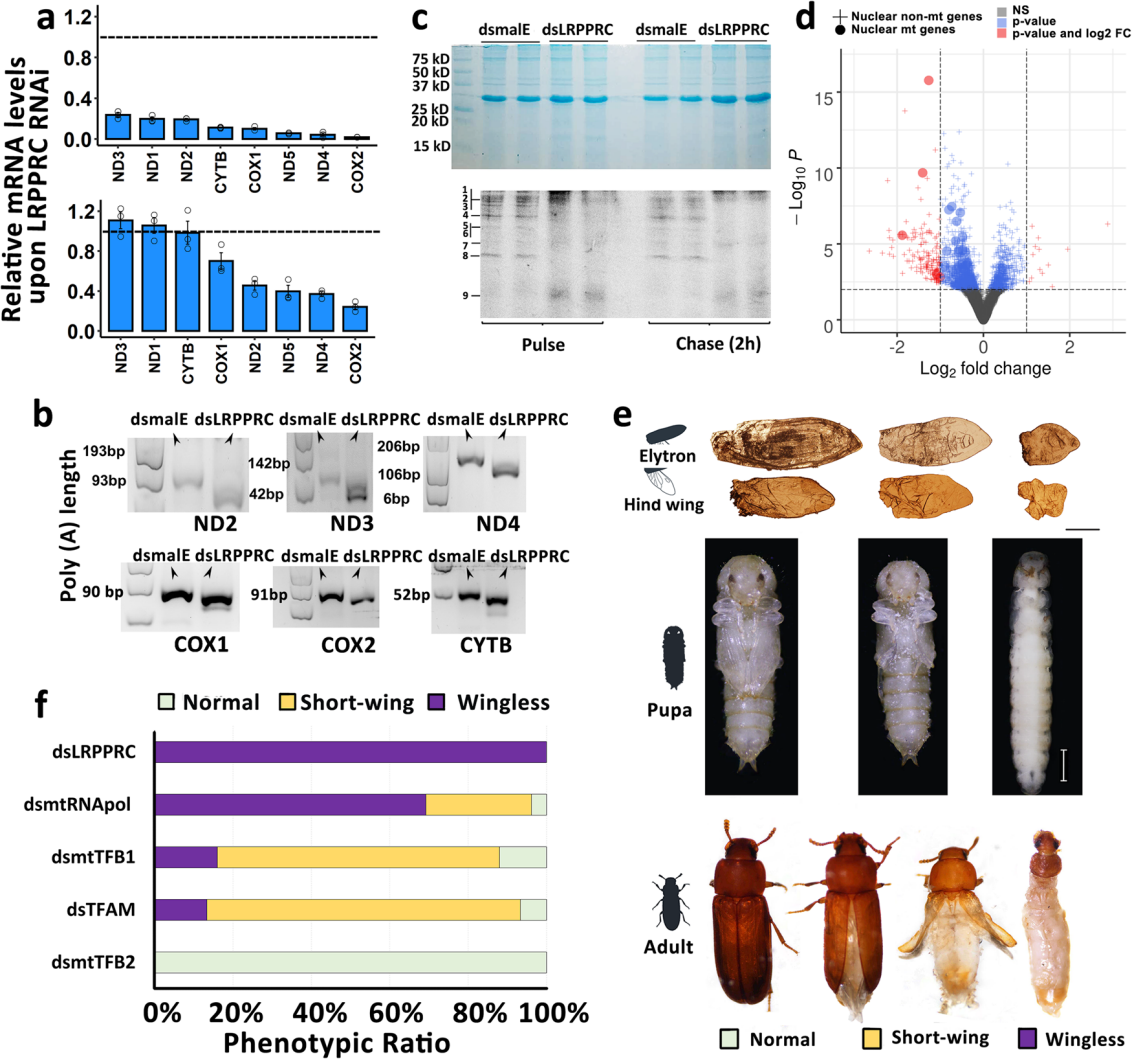
Effect of mitochondrial dysfunction on *T. castaneum* nuclear gene expression and wing development. **a** Relative mRNA levels in *dsmalE* or *dsTcLRPPRC* treated insects were determined by RT-qPCR and normalized using *TcRP49* gene mRNA levels. The expression levels of genes in control *dsmalE*-treated insects was set at 1.0. The data shown are means ± SE (*N* = 3, biological replicates). (Top) Only wing discs were used to extract total RNA. (Bottom) Whole body except wing discs were used to extract total RNA. **b** Polyadenylation profile of ND2, ND3, ND4, COX1, COX2, and CYTB mRNAs in control or *TcLRPPRC* knockdown prepupal wing discs. Total RNA extracted from wing discs was ligated with a DNA adaptor and then reverse-transcribed using an anti-adaptor primer. The length of poly(A) tail was determined by PCR amplification using anti-adaptor and a specific upstream primer of each gene. The first lane shows the position of DNA bands from the 1 kb ladder used to estimate the size of amplified nucleic products representing the length poly(A) tail. ND; mitochondrially encoded NADH:ubiquinone oxidoreductase core subunit. COX; cytochrome c oxidase. CYTB; cytochrome b. **c** De novo mitochondrial translation was determined by ^35^S-methionine incorporation, normalized by total mitochondrial protein stained with Coomassie blue. 1-ND5, 2-COX1, 3-ND4, 4-CYTB, 5-ND1, 6-ND2, 7-ATP6, 8-ND6, 9-ND3. **d** Volcano plot showing differentially expressed genes for larvae with *TcLRPPRC* knockdown relative to control at 24 h after injection. The blue dots indicate significantly differentially expressed genes (DEG) with p-adj <0.05 and less 2-fold change. The red dots indicate DEGs with p-adj <0.05 and over 2-fold change. Nuclear genes encoding for mitochondrial proteins are displayed with filled cycles. **e** Pictures of wings (Top), pupae (Middle), and adults (Bottom) after knocking down mitochondrial gene expression regulators. The cuticle of the insects with severe phenotypes were peeled off to exhibit inner structure. Scale bar of wings = 200 µm. Scale bar of insects = 1000 µm. **f** The ratio of phenotypes of insects injected with dsRNAs of *LRPPRC* (*N* = 60), *mtRNApol* (*N* = 26), *mtTFB1* (*N* = 25), TFAM (*N* = 30) and *mtTFB2* (*N* = 20). *N* = X indicates biologically independent animals.

To confirm that the ecdysis failure and wingless morphology are due to mitochondrial dysfunction induced by the knockdown of LRPPRC, we studied the function of TcLRPPRC. We hypothesized that TcLRPPRC regulates poly(A) length, stability, and translation of mt-mRNA, similar to its counterpart in mammals and fruit flies^25,27^. Determination of relative mt-mRNA levels showed that the abundance of all tested mt-mRNAs decreased 80–100% in wing discs dissected from *dsTcLRPPRC*-treated larvae compared to that in wing discs from control larvae treated with *dsmalE* (Fig. 2a). The decrease in mt-mRNA levels was lower (0–80%) in the other tissues of *dsTcLRPPRC* treated larvae (Fig. 2a). Meanwhile, mt-DNA levels were not significantly different between *dsTcLRPPRC* and *dsmalE* treated insects suggesting that reduction in mt-mRNA levels is not due to reduced mitochondrial density (Supplementary Fig. 2a). The effect of *TcLRPPRC* knockdown on mRNA poly (A) tail length was also investigated. The results showed that all eight mt-mRNAs tested had shorter poly (A) tails in *dsTcLRPPRC* treated larvae compared to that in *dsmalE* treated larvae (Fig. 2b). The investigation of mt-mRNA stability using TcA cells developed from *T. castaneum* showed that degradation of mt-mRNAs increased in *dsTcLRPPRC* treated cells compared that in *dsmalE* treated cells (Supplementary Fig. 2b). Besides, we also observed a significant suppression of de novo mitochondrial translation after knocking down *TcLRPPRC* (Fig. 2c). RNAseq study was conducted to identify the global response of *T. castaneum* to *TcLRPPRC* knockdown. Total RNAs were isolated at 24 and 48 h after injection of *dsTcLRPPRC* and *dsmalE* into newly molted last instar larvae. Sequencing of RNA followed by differential gene expression analysis identified 817 downregulated and 527 upregulated genes with a *p*-value adjusted (padj) ≤0.05 at 24 h in *dsTcLRPPRC* treated larvae when compared to that in *dsmalE* treated control larvae (Fig. 2d, Supplementary Data 1). Interestingly, 74 nuclear genes coding for mitochondrial proteins were significantly enriched in downregulated genes (Fig. 2d and Supplementary Fig. 2c). KEGG module enrichment analysis of downregulated genes identified leucine degradation, beta-oxidation, gluconeogenesis, and citrate cycle as the pathways that are most affected by knocking down *TcLRPPRC* (Supplementary Fig. 2d). However, the downregulation of nuclear genes coding for mitochondrial proteins is not sustained as only six of them were still downregulated at 48 h after *dsTcLRPPRC* treatment (Supplementary Fig. 2e, Supplementary Data 2). These data suggested that the phenotypes observed after knocking down *TcLRPPRC* is induced by mitochondrial function.

### Mitochondrial dysfunction induces wingless or short-wing phenotypes

Other mitochondrial regulators in the mitochondrial transcription initiation complex were knocked down to further confirm the role of mitochondria in *T. castaneum* development^30^. As expected, similar phenotypes with *TcLRPPRC* knockdown were observed (Fig. 2e, f). About 70% of insects injected with *dsmtRNApol* (*mitochondrial DNA-directed RNA polymerase* dsRNA) showed the same phenotypes detected in *TcLRPPRC* knockdown insects (Fig. 2f). Interestingly, short-wing phenotype without ecdysis defects was observed after the knockdown of several mitochondrial regulators (Fig. 2e). After *TFAM* knockdown, most of the pupae showed a short-wing phenotype and were able to eclose to adults (Fig. 2f). *mtTFB2* (*mitochondrial transcription factor B2*) knockdown did not cause any wing development defects, while knockdown of *mtTFB1* (*mitochondrial transcription factor B1*), which is reported to interact with mitochondrial rRNA induced both wingless and short wing phenotypes^31^ (Fig. 2f). The reduction of wing length was caused by the changed wing-body allometry, and no significant difference was observed in the body size or weight in animals with mitochondrial dysfunction (Supplementary Fig. 3). The variability of phenotypes induced by knockdown of different mitochondrial regulators could be related to their importance in maintaining mitochondrial function. The knockdown of *TcLRPPRC* induced the most severe phenotypes, likely because TcLRPPRC regulates both mt-mRNA abundance and translation. These data suggest that there may be thresholds of mitochondrial activity required for larval development. The severe mitochondrial dysfunction caused by *TcLRPPRC* knockdown induced the failure of ecdysis and blocked wing development. In comparison, a moderate mitochondrial dysfunction induced by the knockdown of components of the mitochondrial transcription complex did not affect ecdysis but partially affected wing development and resulted in short wing phenotype. RT-qPCR analysis showed *TcLRPPRC*, *mtRNApol*, *TFAM*, and *mtTFB1* genes had a similar tissue-specific expression pattern, the overall highest expression in the fat body and a peak expression in the wing pads at the beginning of wing growth, which indicates the functional similarity of them (Supplementary Fig. 4). These data indicate an essential role for mitochondria in *T. castaneum* wing growth and demonstrate that varying mitochondrial dysfunction affects the final status of wings.

### IIS pathway regulates *T. castaneum* wing growth by affecting mitochondrial transcription through TFAM

**Fig. 3:**
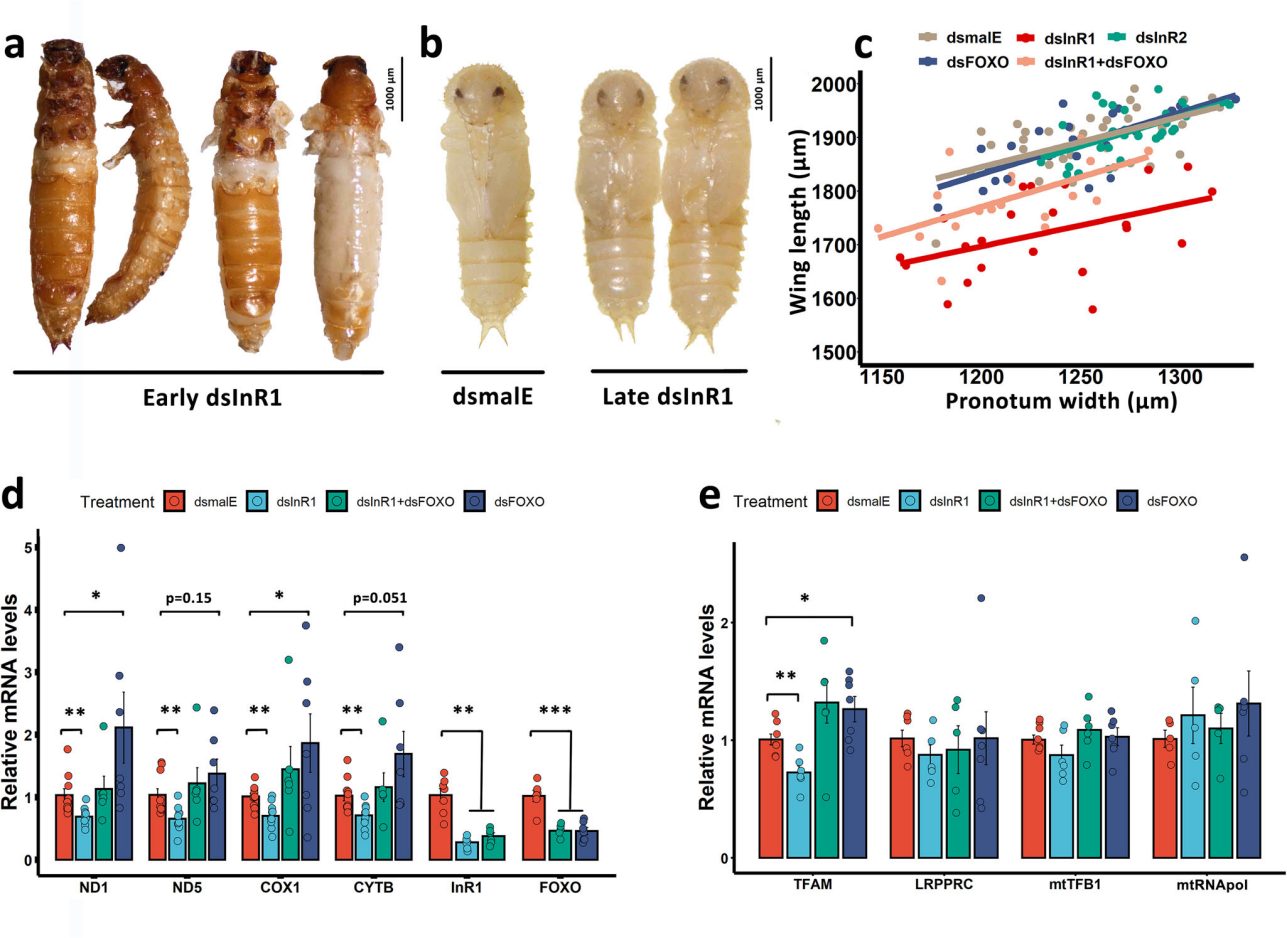
IIS-FOXO regulates *T. castaneum* wing growth through modulation of mitochondrial activity. **a** Pictures of insects with early dsRNA injection. *dsInR1* was injected into the newly molted last instar larvae. Insects were sampled on the 13th day after injection. The developing insects are stuck inside cuticles (the left two insects), the cuticles were peeled off to show the developing insects inside the cuticles (the right two insects). Early injection of *dsInR1* blocked the ecdysis during pupation. Scale bar of insects = 1000 µm. **b** Pictures of insects with late dsRNA treatment. *dsInR1* was injected into the two-day-old last instar larvae. Late treatment of *dsInR1* did not prevent pupation but leads to short wings. **c** Phenotypes of insects treated with IIS key regulators dsRNAs are shown as scatterplots of wing length against body size (pronotum width). The color of dots and regression lines indicates different treatments. *dsmalE* (*N* = 36) and *dsInR2* (*N* = 34) were injected into newly molted last instar larvae. *dsInR1* (*N* = 22) was injected into the two-day-old last instar larvae to acquire pupation. *dsFOXO* was injected to *dsInR1* treated larvae (*N* = 19) or wildtypes (*N* = 23) at the following day after *dsInR1* injection (day three). *N* = X indicates biologically independent animals. **d** RT-qPCR analysis of mitochondrial gene expression. **e** RT-qPCR analysis of mitochondrial regulators gene expression. The data shown are means ± SE. Each dot indicates one biological replicate.

IIS is one of the key pathways involved in the regulation of exaggerated trait growth and wing polymorphism in insects^1,2,5,20–22^. Knockdown of genes coding for IIS pathway components can significantly reduce the size of beetle horns and weapons^1,2,6^. The high population density of the pea aphid *Acyrthosiphon pisum*, inhibits the expression of a miRNA targeting an ABC transporter activating IIS pathway and inducing long-wing morphs^7^. These data and our results inspired us to investigate if mitochondria are involved in wing developmental plasticity mediated by IIS pathway. We first examined if IIS signaling regulates *T. castaneum* wing growth. Two Insulin receptors (InR) were identified in *T. castaneum* genome^32^. Previous studies showed that InR1 is essential for larval development, and the function of InR2 in larval development is redundant^12,32^. Knockdown of *InR1* by injection of *dsInR1* into early (day 0) last instar larvae caused the failure of ecdysis and pupation (Fig. 3a). When control insects became adults, half of the *dsInR1* injected insects developed adult structures without ecdysis, and two pairs of retarded wings were found after peeling off old cuticles (Fig. 3a). While injection of *dsInR1* into last instar larvae at a later stage (day 2) did not fully block pupal ecdysis, the pupae developed from *dsInR1* injected larvae showed short wings (Fig. 3b, c). FOXO, the main downstream regulator of IIS pathway, is shown to regulate wing morphs^5^. In the brown planthopper, *N. lugens*, knockdown of *InR1* induced short-wing morph, but injection of both *InR1* and *FOXO* dsRNAs converted the short-wing to long-wing morph^5^. In our studies, injection of *dsFOXO* one day after *dsInR1* injection partly rescued wing growth (Fig. 3c). These data suggest that IIS-FOXO signaling is conserved in regulating disproportionate wing growth in *T. castaneum*. To further determine if IIS signaling functions through mitochondria to regulate wing growth, RT-qPCR was used to measure mRNA levels of mitochondrial genes. Results showed that knockdown of *InR1* suppressed the mitochondrial gene expression, while injection of both *dsInR1* and *dsFOXO* rescued the expression of mitochondrial genes (Fig. 3d). This corresponds with the phenotypes observed above, which indicate mitochondrial activity indeed acts downstream of IIS signaling. In humans, mitochondria function was found to interact with insulin signaling^18,33^. Mitochondrial dysfunction was reported to interact with insulin resistance, and insulin signaling enhances mitochondrial gene expression and protein synthesis^18^. *FOXO3a*, a homolog of *FOXO* in humans, inhibits mitochondrial gene expression through downregulating mitochondrial regulators^34^. We tested if *InR1* knockdown affects the expression of mitochondrial regulators. *TFAM* expression was suppressed after knockdown *InR1*, and the suppression was rescued with the coinjection of *dsFOXO* (Fig. 3e). These suggest that *TFAM* is regulated by IIS-FOXO. Other mitochondrial regulation factors tested did not show a significant difference in expression after knockdown *InR1* or *FOXO* (Fig. 3e). In addition to TFAM, other uncharacterized mitochondrial regulators could also be involved in the regulation of mitochondrial activity tuned by IIS. Further studies are required to identify these regulators. Mitochondria are unlikely to affect wing growth through IIS pathway because most of IIS signaling genes did not show significant change after *dsLRPPRC* induced mitochondria dysfunction (Supplementary Fig. 5). In addition, knockdown of mitochondrial regulators induced more severe wing deficiency compared with insects with *InR1* knockdown (Figs. 2e and 3a).

### Mitochondria regulate wing growth through wing-patterning genes

**Fig. 4:**
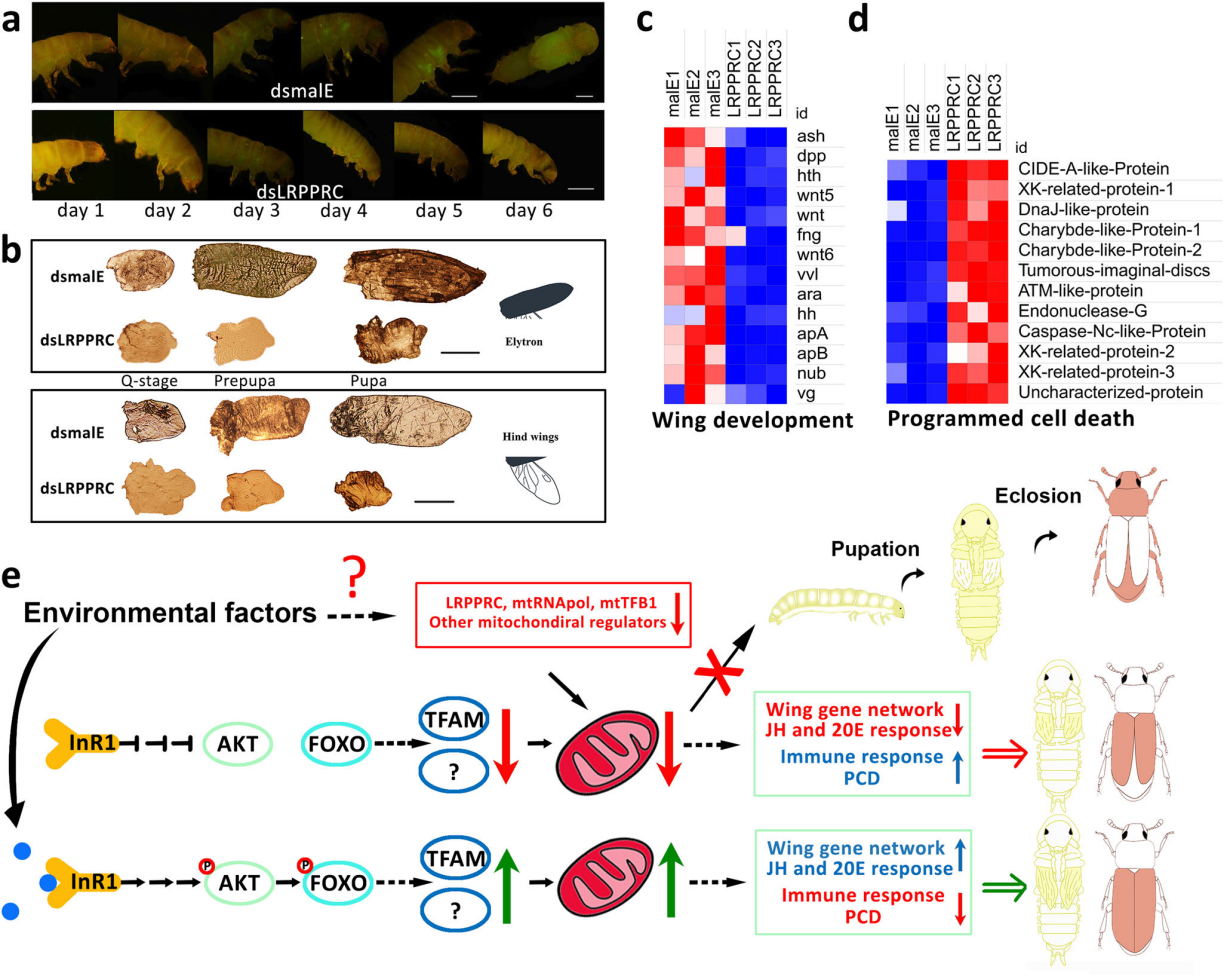
Mitochondrial dysfunction repressed genes in the wing-patterning network. **a** *pu11* last instar larvae treated with *dsmalE* or *dsTcLRPPRC* and photographed under a fluorescence microscope from day 1 to day 6. Fluorescence increased during the larval development in the control larvae. Fluorescence began to appear on day 3 but decreased and disappeared by day 5 in *dsTcLRPPRC* treated larvae. Scale bar = 1 mm. **b** Pictures of elytron (Top) and hind wings (Bottom) from *malE* or *TcLRPPRC* dsRNA injected *T. castaneum* during quiescent, prepupal and pupal stages. Scale bar = 200 µm. **c** Heatmap showing the differentially expressed genes involved in insect wing development. *T. castaneum* orthologs identified by searching *T. castaneum* genome using *D. melanogaster* gene sequences. ash, achaete scute complex protein; dpp, decapentaplegic; hth, homothorax; wnt, wingless/integrated; fng, fringe; vvl, ventral veins lacking; ara, araucan; hh, hedgehog; apA, apterous A; apB, apterous B; nub, nubbin; vg, vestigial. **d** Heatmap showing the genes in programmed cell death are upregulated in prepupal wing discs upon knockdown of *TcLRPPRC*. Gene ids of these genes are listed in Supplementary Table 2. **e** Schematic model for regulation of IIS to wing plasticity mediated by mitochondria. Arrows and bars indicate activation or suppression of gene expression. Dashed lines denote unclear mechanisms. *T. castaneum* does not show wing polyphenism in nature. This model uses hypothetical insect images to show  the role of mitochondria in regulating insect developmental plasticity.

Another interesting question raised by these studies is how mitochondrial dysfunction regulates wing development. Seeking an explanation, wing development was tracked in insects treated with *dsTcLRPPRC* or *dsmalE* using *pu11* transgenic *T. castaneum* line that expresses EYFP in wing primordium^35,36^. In control insects, fluorescent wing discs were first detected on the 3rd day of the last instar larval stage and the wings were completely developed by ecdysis to the pupal stage (Fig. 4a). The knockdown of *TcLRPPRC* did not affect the initiation of wing disc cell proliferation. Fluorescence in the wing discs of larvae treated with *dsTcLRPPRC* was detected at the same time (day 3) as in control larvae; but unlike in control larvae, the fluorescence did not increase on day 4 and eventually disappeared by day 6 (Fig. 4a). The wing discs from control quiescent (Q-stage), prepupal and pupal stage insects were dissected and photographed. The wing discs from control larvae showed normal growth and differentiation to elytron and hind wings (Fig. 4b). In contrast, the wing discs from larvae treated with *dsTcLRPPRC* started proliferation but did not differentiate into elytron and hind wings (Fig. 4b). To study mitochondrial function in the regulation of wing growth, RNA was isolated from wing discs of prepupae from insects treated with *dsTcLRPPRC* or *dsmalE* and sequenced. Differential gene expression analysis of RNA sequences identified 1145 upregulated and 1274 downregulated genes with a ≥2-fold change and padj ≤ 0.05 in *dsTcLRPPRC* treated insects when compared to that in control insects (Supplementary Fig. 6a, Supplementary Data 3). Insect wing-patterning genes are well studied in *D. melanogaster*^37^. *T. castaneum* has highly conserved wing-patterning network genes that regulate the patterning of elytra and hind wings^38^. Genes involved in D-V axis patterning are affected by the knocking down of *TcLRPPRC*. Two *T. castaneum apterous* homologs (*apA*, *apB*), *fringe* (*fng*), three *wingless* (*wnt*) homologs and *achaete-scute homolog* (*ash*) were suppressed in wing discs after knocking down *TcLRPPRC*. While key regulators in A-P patterning, including *decapentaplegic* (*dpp*) and *hedgehog* (*hh*) were also affected (Fig. 4c). Two wing identity genes, *nubbin* (*nub*) and *ventral veins lacking* (*vvl*), were downregulated in *TcLRPPRC*-knockdown wing discs (Fig. 4c). Knockdown of these wing-patterning genes such as *nub*, *vestigial* (*vg*) and *aptA/B* in *T. castaneum* induced short-wing or wingless phenotypes^36,39^. These results suggest that the downregulation of these wing-patterning genes induced by mitochondrial dysfunction may directly cause wing growth deficiency. Besides, genes involved in the action of two most important insect hormones, JH and 20-hydroxyecdysone (20E), such as *Met* (*Methoprene-tolerant*), *Kr-h1* (*Kruppel homolog 1*), *EcR* (*Ecdysone receptor*), and *BrC* (*Broad-complex*) were also downregulated in wing discs from *TcLRPPRC* knockdown larvae, which may have indirectly affected wing development due to their importance in regulating wing disc growth^40,41^(Supplementary Fig. 6b). RNA-seq data also revealed the downregulation of 21 genes coding for pupal cuticle proteins (Supplementary Fig. 6c). Wing discs eventually disappeared in insects injected with *TcLRPPRC* dsRNA, despite the cell proliferation at the initial stages of development. This could have been induced by the upregulation of immune response and programmed cell death pathways due to the dysfunction of mitochondria (Fig. 4d and Supplementary Fig. 6d). Gene ontology enrichment analysis identified transmembrane transport, macromolecule metabolic process and carboxylic acid biosynthetic process are enriched in downregulated genes (Supplementary Fig. 7). The upregulated genes are found to be involved in the carbohydrate catabolic process, defense response, intracellular organelle, and cell adhesion molecule binding (Supplementary Fig. 8).

## Discussion

Insects undergoing both hemimetabolous and holometabolous metamorphosis have evolved wing polyphenism to rapidly acclimatize to heterogeneous environments. The molecular and developmental mechanisms governing wing growth plasticity and widespread phenotypic plasticity are poorly understood. Recent milestone findings, including the effect of ecdysone on pea aphid (*A. pisum*) transgenerational wing dimorphism and IIS/FOXO pathway on brown planthopper (*N. lugens*) wing dimorphism, start to shed light on the molecular mechanisms^5,42^. Both aphids and planthoppers are from the order Hemiptera undergoing hemimetabolous metamorphosis. For hemimetabolous insects, wings start to rapidly grow and develop from wing buds during the last nymph instar, while for the holometabolous, wing discs start exponential growth and development during the late last instar larval stage. Insect wings are the representative adult structure of insects. The growth of wings typically signifies the start of insect metamorphosis^8^. Multiple events, such as degeneration of larval tissues and development of pupal and adult structures, occur during metamorphosis. How energetic and metabolic dynamics cope with massive organ remodeling during metamorphosis has been asked and studied by biologists for decades. Previous studies on the energetic pattern of insect metamorphosis reported a U-shaped metabolic curve during the pupal development of insects. The metabolic rate declines sharply at the beginning of metamorphosis and remains low until before eclosion into adults^43,44^. This makes sense as insects need to conserve energy during the pupal stage for the active adult stage because insect adults do not usually intake much food but require much more energy for reproduction^43^. In this study, we investigated the role of mitochondria in insect metamorphosis. Our study in holometabolous insect *T. castaneum* showed that mitochondrial dysfunction is not essential for most tissue remodeling and adult structure formatting except for wings. This result corroborates the reported U-shaped metabolic curve with an extremely low metabolic rate during most of the pupal stage. In addition to the energetic pattern, the redundancy of mitochondria during metamorphosis can also be supported physiologically; the tracheal system undergoes remodeling during pupal development, and the anterior and posterior parts of the dorsal trunks are collapsed and filled with fluid until right before eclosion^45^ affecting oxygen delivery. Hypoxic conditions did not affect the overall metabolic curve and the whole metamorphosis progress. The insects treated with anoxia are nearly the same as control insects except for several hours of delay^43^. The maximum growth in holometabolous insects occurs during the last instar larval stage. After body weight reaches a critical developmental checkpoint called the threshold size, metamorphosis is started by downregulation of JH-response genes such as *Kr-h1* and upregulation of pupal and adult genes such as *Br-C* and *E93* (*Ecdysone-induced protein 93*)^46^. We measured the expression levels of these metamorphosis regulators, including *Kr-h1*, *Br-C*, and *E93*, and no significant effect was found on the expression of these genes by mitochondrial dysfunction, indicating that the timing of larval growth and the start of metamorphosis does not change in last instar larvae with mitochondrial dysfunction (Supplementary Fig. 9a). Taken together, these data suggest that final body size and tissue remodeling during metamorphosis are not significantly affected by mitochondrial dysfunction.

However, wing growth, unlike other parts of metamorphosis, is significantly affected by mitochondrial dysfunction (Fig. 2e). Wing growth in holometabolous insects usually starts after the body reaches critical size and stops growth^8^. How insects subsequently develop wings accurately to match the body size is an interesting question and may hold the key to understanding wing polyphenism. Previous studies showed that the timing of release and levels of ecdysteroids regulate cell division that determines the final wing size in *D. melanogaster*^8^. Insect hormones are essential for wing development in insects^40,41,47^. By knocking down the receptors of insect hormones, JH and 20E, we show that ecdysone and JH also regulate wing growth in *T. castaneum* (Supplementary Fig. 9b). Our studies suggest that mitochondrial activity may be required for insect wing growth mediated by insect hormones during metamorphosis since mitochondrial dysfunction significantly suppresses the expression of JH and 20E response genes in wing discs (Supplementary Fig. 6b). Mitochondria are involved in multiple important functions, including serving as a powerhouse of the cell, calcium homeostasis maintenance, generation of ROS, apoptosis, and metabolism^17^. In this study, we tried to confine the definition of mitochondrial dysfunction into reduced mitochondrial activity through suppressing mitochondrial genome-encoded gene transcription and translation. However, reduced mitochondrial activity may also induce other cellular changes listed above; thus, how mitochondrial dysfunction induced organ failure should be studied in the future. Studies on the function of mitochondria in insect wing development are rare except in *D. melanogaster* using wing imaginal discs as a model to study developmental and cell biology. Previous studies using *D. melanogaster* mutants of mitochondrial proteins showed that specific mitochondrial proteins are required for both cell cycle and cell growth of wing discs^48,49^. These results indicate that cell proliferation is inhibited in wing imaginal discs by mitochondrial dysfunction, which may eventually induce the failure of wing development. However, in this study, we did not observe morphologically adverse effects on the development of imaginal discs of antenna, compound eyes, and genitalia other than wings (Figs. 1a and 2e). Moreover, genes involved in the cell cycle are not significantly enriched in wing discs differentially expressed genes (DEG) (Supplementary Fig. 7). Thus, although mitochondria are essential for cell proliferation, they may not be the key cause of blocked wing growth. Based on our current knowledge, we suggest that mitochondria affect wing growth and development by suppressing the expression of wing-pattern genes and those responding to insect hormones, including JH and 20E. From the view of wings, we found a surged mitochondrial transcription activity during wing growth in the late last larval stage (Supplementary Fig. 4e). Thus, an increased mitochondrial activity should be required for activation of these growth signaling, including insect hormones and wing-pattern genes in wing discs. Nevertheless, wing growth signaling may require different mitochondrial activity thresholds since two different types of wing phenotypes were observed, a severe one with failure of cellular differentiation and an intermediate one with shorter elytron and hind wings, suggesting that pathways regulating cell differentiation require a high mitochondrial activity, while wing growth require a moderate mitochondrial activity. More studies are required in the future to address how mitochondrial activity precisely interacts with these growth factors.

We also showed that IIS/FOXO could regulate mitochondrial activity by tuning TFAM, indicating that mitochondria are involved in IIS-mediated insect wing polyphenism. Our conclusion is supported by a recent transcriptome study using long and short wing planthopper morph wing buds^16^. Zhang et al. showed that metabolism pathways, especially for those in mitochondria, including amino acid and fatty acid metabolism, are highly enriched in DEGs by KEGG analysis, and interestingly the nuclear genes encoding for mitochondrial proteins are also significantly enriched in DEGs (6.4% (19/299) enrichment in *N. lugens* DEGs vs. overall 2.6% (385/14972) mitochondrial proteins in *T. castaneum* genome, chi-square statistic *p* value = 0.000112)^16^. Besides, mitochondria may also be involved in regulating the conditional growth of beetle weapons. Okada et al. recently showed that insulin peptide ILP2, specifically regulates broad-horned flour beetle *Gnatocerus cornutus* mandible growth, while ILP2 was highly expressed in the fat body of large larvae with a better nutritional status^2^. Fat body is one of the tissues that have the highest mitochondrial activity in insects (Supplementary Fig. 4e), which raised the potential that ILP2 may interact with mitochondrial activity to regulate mandible growth. Target of rapamycin (TOR) is another conserved nutritional sensing pathway in animals. Previous studies showed that silencing of *TOR* also induced a smaller wing phenotype in *T. castaneum*^50^. TOR can regulate mitochondrial oxidative function, which indicates that TOR likely regulates wing growth through mitochondria. Mitochondrial function has been reported to be directly affected by environmental cues or other cellular signaling in addition to IIS pathway, which raises an interesting hypothesis that other signaling pathways yet to be discovered may regulate insect wing polyphenism independent of IIS/FOXO pathway by acting on mitochondrial activity directly^51^.

One potential concern of this study is that *T. castaneum*, the model insect we used, does not show an obvious wing polyphenism in nature. However, recent advances suggested that those conserved gene networks are more likely co-opted in the formation of new traits or evolution of developmental plasticity other than evolutionary innovated genes^52–54^. For example, Hu et al. showed that the wing gene regulatory network is required for the development of many insect appendages and outgrowths, including beetle horns^52^. Casasa et al., using genome-wide transcription data in three closely related species of horned beetles, demonstrated that conserved genes are more critical in developmental regulation and the evolution of nutritional plasticity^53^. Thus, our results are valuable in understanding the mechanism of developmental plasticity. Here, our study suggested that mitochondria, the oldest undisputedly “pal” of eukaryotic organisms, may have been utilized by animals for the regulation of developmental plasticity to help adapt to nature (Fig. 4e).

## Methods

### Insect Rearing

GA-1, a standard laboratory strain collected from a Georgia corn bin, and *pu11* line were used in these studies^35,55^. Insects were reared on organic wheat flour mixed with 5% blended yeast in complete darkness at 30 °C and 60 ± 5% relative humidity.

### dsRNA synthesis and microinjection

Primers containing T7 promoter sequence at the 5’ end designed at E-RNAi site (https://www.dkfz.de/signaling/e-rnai3/) were used to amplify 150–500 bp fragments of target genes (Supplementary Table 1)^56^. dsRNAs were synthesized using the MEGAscript RNAi Kit (Invitrogen, Carlsbad, CA) following the manufacturer’s protocol. dsRNAs were purified by phenol/chloroform and precipitated by sodium acetate/ethanol. Then the dsRNAs were quantified and diluted to 1–3 μg/μl concentration, and 200 nl of dsRNA solution was injected into newly ecdysed last instar larvae using Nanoject III programmable nanoliter injector (Drummond Scientific Company, Broomall, PA). The dsRNA prepared from 800 bp bacterial *malE* gene was used as a control.

### Scanning electron microscopy

To identify insect cuticles, insects at 13 days after injection with *dsTcLRPPRC* were collected. Wild-type larvae, pupae, and adults were also collected. The old cuticles on the pronotum of insects treated with *dsTcLRPPRC* dsRNA were broken with fine forceps to expose the inside cuticle. The methods used for preparing samples and examination under a scanning electron microscope were previously described^57^. Insects were collected and fixed in 2.5% glutaraldehyde overnight. Then the samples were dehydrated by incubating with 30%, 50%, 70%, 80%, 90%, 95%, and 100% ethyl alcohol for 10 min in each concentration. The samples were dried at EM CPD300 critical point dryer (Leica, Wetzlar, Germany) and coated with platinum by using Leica ACE 600 carbon/sputter coater. Quanta 250 (FEI, Hillsboro, OR) was used to capture the images.

### Muscle staining and confocal microscopy

Prepupae treated with *dsmalE* or *dsTcLRPPRC* were collected and the first and second abdominal segments were cut using the micro scissors. The fat body and alimentary canal were removed using forceps, and the integument was cut along sides to separate ventral and dorsal parts. Then the integuments were incubated in Ex-cell 420 medium (Sigma-Aldrich, St. Louis, MO) containing MitoView™ 650 Mitochondria staining Dye (Biotium, Fremont, CA) for 15 min. The tissues were then mounted on a slide and examined under Leica SP8 DLS using 638 nm excitation filter.

### RNA isolation, cDNA synthesis, and quantitative reverse transcription PCR (RT-qPCR)

Total RNA was isolated using TRI reagent (Molecular Research Centre Inc., Cincinnati, OH). RNA concentrations were measured using Nanodrop 2000 (Thermos Fisher, MA, USA). The cDNAs were synthesized using M-MLV Reverse Transcriptase (Invitrogen, Carlsbad, CA) according to the manufacturer’s instruction. The relative mRNA levels were determined by RT-qPCR in StepOnePlus™ Real-Time PCR System using *TcRP49* as a reference gene.

### Poly(A) tail length measurement

Measurement of mitochondrial mRNA Poly(A) tail length was performed as described previously with slight modifications^58^. In brief, 2 μg total RNA was ligated with a linker DNA oligonucleotide using T4 RNA Ligase (New England Biolabs). RNA was then extracted by phenol/chloroform and precipitated by sodium acetate/ethanol. Purified RNA was reverse-transcribed using M-MLV Reverse Transcriptase (Invitrogen, Carlsbad, CA) with an anti-linker primer. The cDNA product was used as the template in a standard PCR reaction using PrimeSTAR GXL DNA Polymerase (Takara) and anti-linker primer and a primer specific to mitochondrial gene (Supplementary Table 1).

### Cell culture and mt-mRNA stability

TcA cell line was derived from *T. castaneum* in the late pupal stage^59^. Cells were maintained in Ex-cell 420 medium (Sigma-Aldrich, St. Louis, MO) with 10% Fetal Bovine Serum (FBS, VWR Seradigm, Radnor, PA) at 28 °C. TcA cells were seeded in 12-well plates and treated with *TcLRPPRC* or *malE* dsRNA. TcA cells were collected and used to measure mt-mRNA stability at 48 h after treatment following the methods described previously with slight modifications^60^. The cells were treated with 1 μg/ml ethidium bromide (EtBr) to stop mitochondrial transcription. At 0, 2, 4, and 6 h after treatment with EtBr, the cells were collected into TRI reagent (Molecular Research Centre Inc., Cincinnati, OH). The mRNA levels were determined as described above.

### De novo translation

Prepupae treated with *malE* and *TcLRPPRC* dsRNA were collected to prepare mitochondria as described previously with modifications^61^. Insects were first washed by phosphate-buffered saline (PBS) buffer and transferred into 2 ml standing screw cap tubes (Fisher Scientific). 1 ml ice-cold IBc buffer (Buffer for cell and mouse liver mitochondria isolation; 10 ml 0.1 M Tris–MOPS and 1 ml of EGTA/Tris were added to 20 ml 1 M surcose, then distilled water was added to make up 100 ml IBc buffer. pH was adjusted to 7.4) was added to the tube. The insects were homogenized using FastPrep-24^TM^ 5G (MP Biomedicals) with speed as 6 m/s for 10 s. The tubes were transferred to ice immediately after homogenization. Then, the homogenate was centrifuged at 600 × *g* for 10 min at 4 °C. The supernatant was transferred to pre-cold Eppendorf tubes and centrifuge at 7000 × *g* for 10 min at 4 °C. The precipitated pellet was washed once and resuspended using ice-cold IBc buffer. Concentration of mitochondria was assessed by measuring protein concentration using A280 by Nanodrop 2000. Collected mitochondria were used immediately for de novo translation study. Methods to study de novo translation with [^35^S]-methionine (PerkinElmer, Waltham, MA) were conducted as reported previously^62^. Mitochondria were aggregated at 7000 × *g* for 5 min at 4 °C and resuspended in 1.5 ml translation buffer (recipe can be found at Hilander et al., 2018^62^) added with [^35^S]-methionine. Reactions were incubated 1 h at 37 °C with 300 rpm shaking. Then, the reaction tubes were centrifuged at 7000 × *g* for 5 min at 4 °C, and the supernatant was discarded. Pellet was resuspended with 1 ml translation buffer. The suspension was split into two tubes. One tube was used as “pulse” by adding loading dye and stored in −20 °C. Another tube was used as “chase” by adding cold methionine (final concentration 60 µg/ml) and incubated at 37 °C for 2 h. The proteins were then separated by SDS-PAGE gel. Then the gel was stained by Coomassie brilliant blue R-250 staining solution (Bio-Rad, Hercules, CA) and dried with a gel drier. Dried gel was exposed on a phosphor-Imager screen for 2 days, and the screen was scanned in a phosphor Imager (Typhoon 9500, GE Healthcare Life Sciences).

### Allometric measurements

Nikon SMZ745T stereoscopic microscope and DS-Fi3 camera were used to measure the size of the pupal wing and thorax. All pupae were laid flat and took the picture at the same scope. For wing length, both left and right elytron were measured, and the mean value was used to represent the size of one individual. The pronotum width was measured as the distance between the edges of the left and right sides.

### RNA sequencing and data analysis

Total RNA was isolated, and the concentrations were determined by Nanodrop 2000 (Thermos Fisher, MA, USA). The RNA was quality checked and the libraries were prepared and sequenced on DNBseq platform with a read length of 100 bp at BGI, Shenzhen, China. Adapters were trimmed, and the quality of data was checked by FastQC. The data were then mapped to *T. castaneum* genome using STAR. Counts per gene were calculated by RSEM. Differential expression analyses were conducted by DESeq2 and filtered for an adjusted *p*-value <0.05 using Benjamini and Hochberg method. Gene ontology enrichment analysis was conducted by ClueGo in Cytoscape^63^. KEGG module enrichment analysis was performed through clusterProfiler in R platform.

### Statistics and reproducibility

Number of replicates is defined in the legend of each figure. Error bars indicate means ± standard error. Two-tailed unpaired Student’s t test was used for the comparisons between single groups. One-way ANOVA with Turkey’s post-test was used to compare multiple groups.

### Reporting summary

Further information on research design is available in the Nature Portfolio Reporting Summary linked to this article.

## Supplementary information

Supplementary Information

Description of Additional Supplementary Files

Supplementary Data 1

Supplementary Data 2

Supplementary Data 3

Supplementary Data 4

Reporting Summary

## Data Availability

The data used to generate the main figures can be found at Supplementary data 4. Raw RNA-seq data can be found at in the National Center for Biotechnology Information under accession BioProject: PRJNA732477. Other raw and analyzed data in this study are available from the corresponding author per request.
