# Supplementary material for: Mitochondria dysfunction impairs Tribolium castaneum wing development during metamorphosis
Source: Commun Biol. 2022 Nov 15;5:1252. doi: 10.1038/s42003-022-04185-z (PMC9666433; doi:10.1038/s42003-022-04185-z)
Supplement: Supplementary file 1 — Supplementary Information [file 42003_2022_4185_MOESM1_ESM.pdf]

# Supplementary Information:

## Mitochondria dysfunction impairs *Tribolium castaneum* wing development during metamorphosis

Yaoyu Jiao and Subba Reddy Palli*

Department of Entomology, College of Agriculture, Food and Environment, University of Kentucky, Lexington, Kentucky 40546, USA

* Corresponding author, Subba Reddy Palli

Phone: 859 257-4962, Fax: 859 323-1120, Email: rpalli@uky.edu

**Author Contributions:** Y.J. and S.R.P. designed research; Y.J. performed research; Y.J. and S.R.P. analyzed data; Y.J. and S.R.P. drafted the paper.

**Competing Interest Statement:** The authors declare no competing interest.

**Keywords:** LRPPRC, developmental plasticity, FOXO, wing polyphenism, mitochondrial transcription initiation complex.

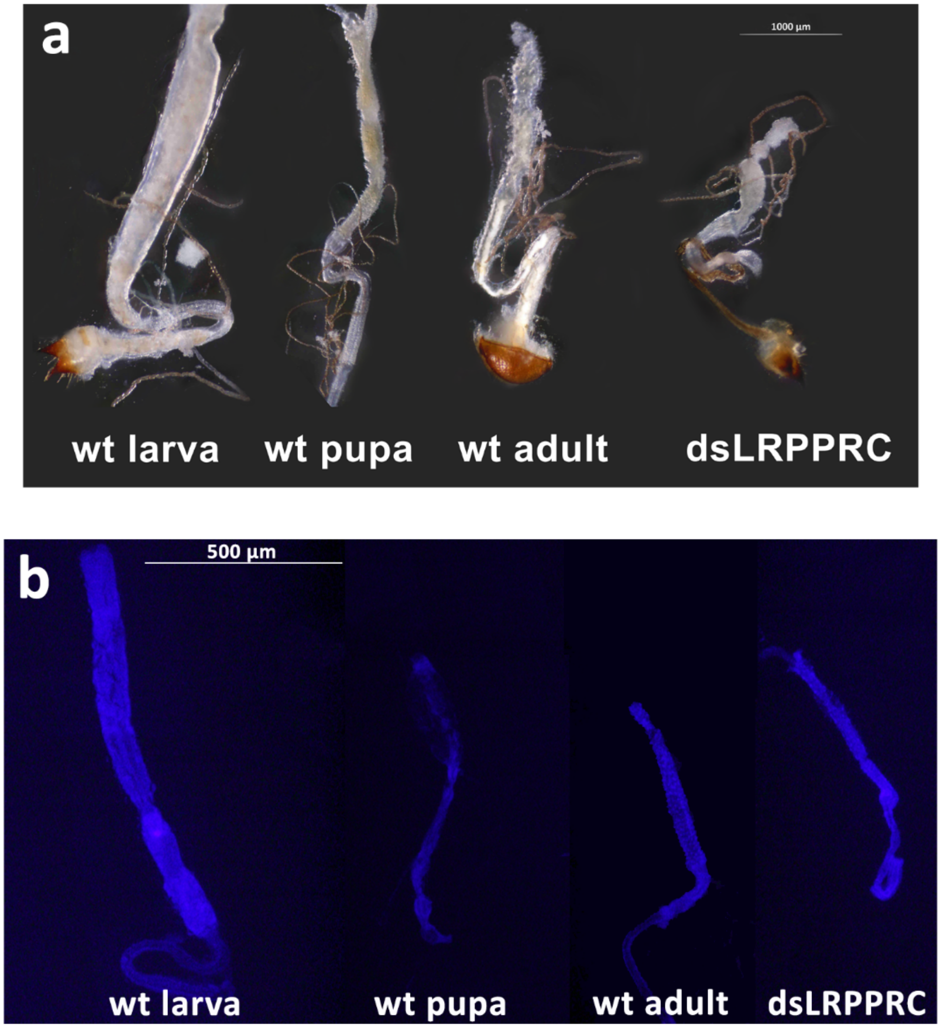


**Supplementary Figure 1.** Alimentary canals dissected from wild-type larva, pupa, adult, and insects treated with *TcLRPPRC* dsRNA after observing sclerotized adult cuticle structures. **a.** Under white light. **b.** The alimentary canals stained with DAPI (4',6-diamidino-2-phenylindole) were photographed using a blue/cyan filter.

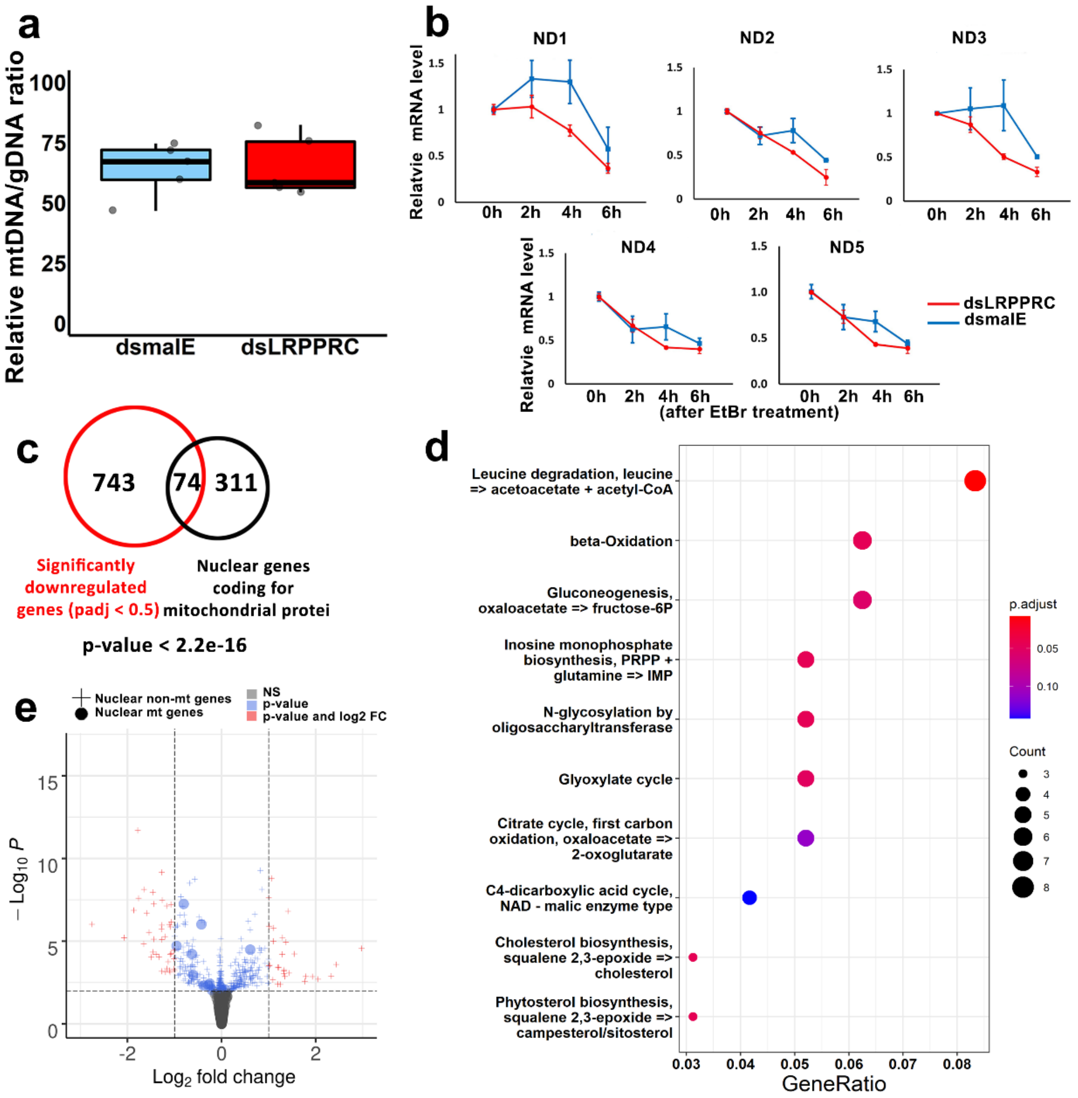


**Supplementary Figure 2.** Effect of *LRPPRC* knockdown on mitochondrial function and gene expression. **a.** Mitochondrial DNA levels were determined by RT-qPCR analysis of genomic DNA using *TcRP49* nuclear gene and *ND2* mitochondrial gene for normalization (N=5). **b.** Decay of mt-mRNAs, ND1, ND2, ND3, ND4 and ND5 in TcA cells treated with *dsTcLRPPRC* or *dsmalE*. Total RNA was extracted at 0, 2, 4 and 6 h after inhibition of mitochondrial transcription and mt-mRNA levels were quantified by RT-qPCR using *TcRP49* gene for normalization. Means ± SE is shown. ND, NADH: ubiquinone oxidoreductase core subunit. **c.** Venn diagram depicting the shared genes among significantly downregulated genes and nuclear genes coding for mitochondrial proteins. Statistical analysis was performed using $\chi^2$ test. **d.** KEGG analysis of down-regulated genes at 24 h after injection. The gene ratio is calculated as k / n (k=gene id overlaps with the gene set, n=size of overlap with the source database). The p-value is correlated with the count and gene ratio to gauge the likelihood of a significant enrichment. **e.** Volcano plot showing differentially expressed genes in *TcLRPPRC* knockdown relative to control larvae at 48 h after the injection. The blue dots indicate significantly differentially expressed genes (DEG) with p-adj < 0.05 and

less 2-fold change. The red dots indicate DEGs with p-adj < 0.05 and over 2-fold change. Nuclear genes encoding for mitochondrial proteins are displayed with filled cycles.

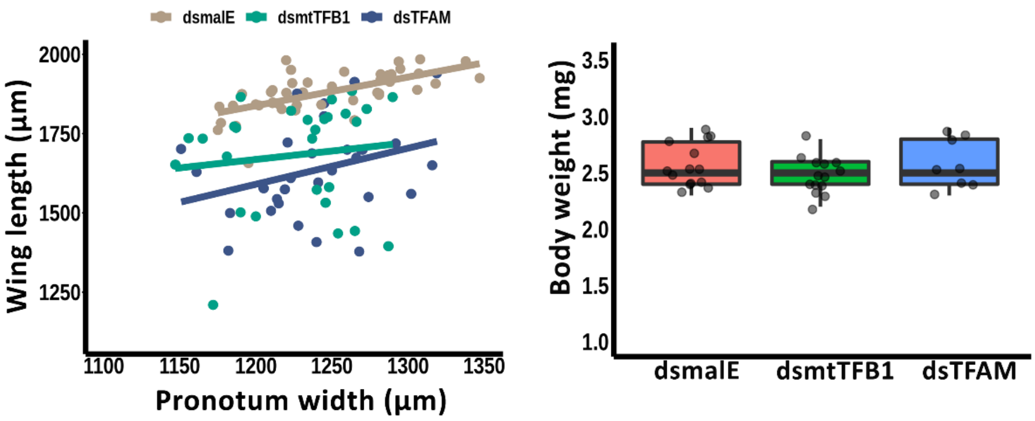


**Supplementary Figure 3.** Effect of mitochondrial dysfunction on body size and weight. **a.** Phenotypes of insects treated with *dsmalE* (N=47), *dsmtTFB1* (N=28) and *dsTFAM* (N=30) are shown as scatterplots of wing length against body size (thorax width). The color of dots and regression lines indicate different treatments. **b.** Body weight of insects treated with *dsmalE* (N=14), *dsmtTFB1* (N=14) and *dsTFAM* (N=8). The color of dots and boxplots indicates different treatments. To eliminate the sexual effect, only 1-day-old males were used to measure body weight.

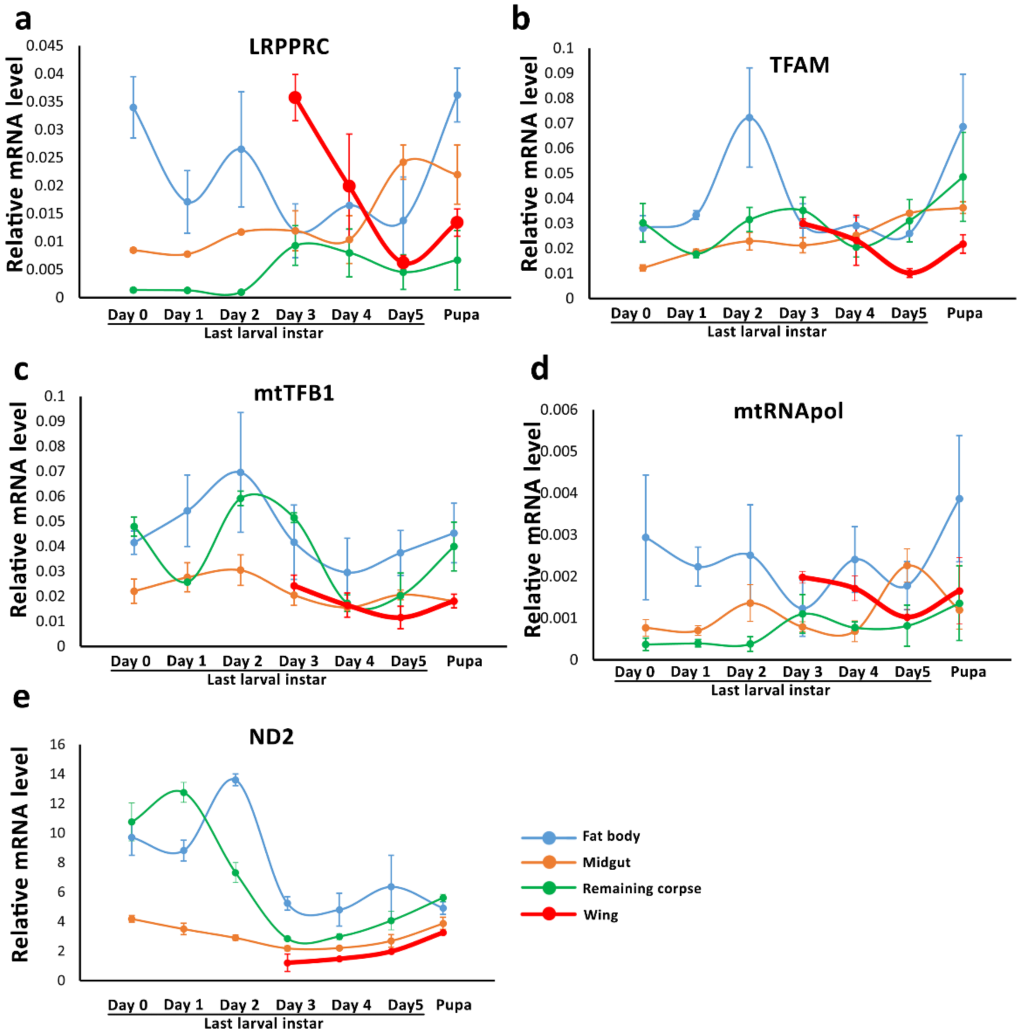


**Supplementary Figure 4.** Tissue-specific expression profile of mitochondrial regulators. **a. - e.** Developmental tissue-specific expression of LRPPRC, TFAM, mtTFB1, mtRNApol and ND2 in *T. castaneum* last instar larvae and newly molted pupae. Fat body, alimentary canal, wing discs and the remaining corpse were dissected from staged insects. Tissues from five insects were pooled and used to extract total RNA. The RNA was converted into cDNA and used to quantify mRNA levels by RT-qPCR. Means ±SE is shown.

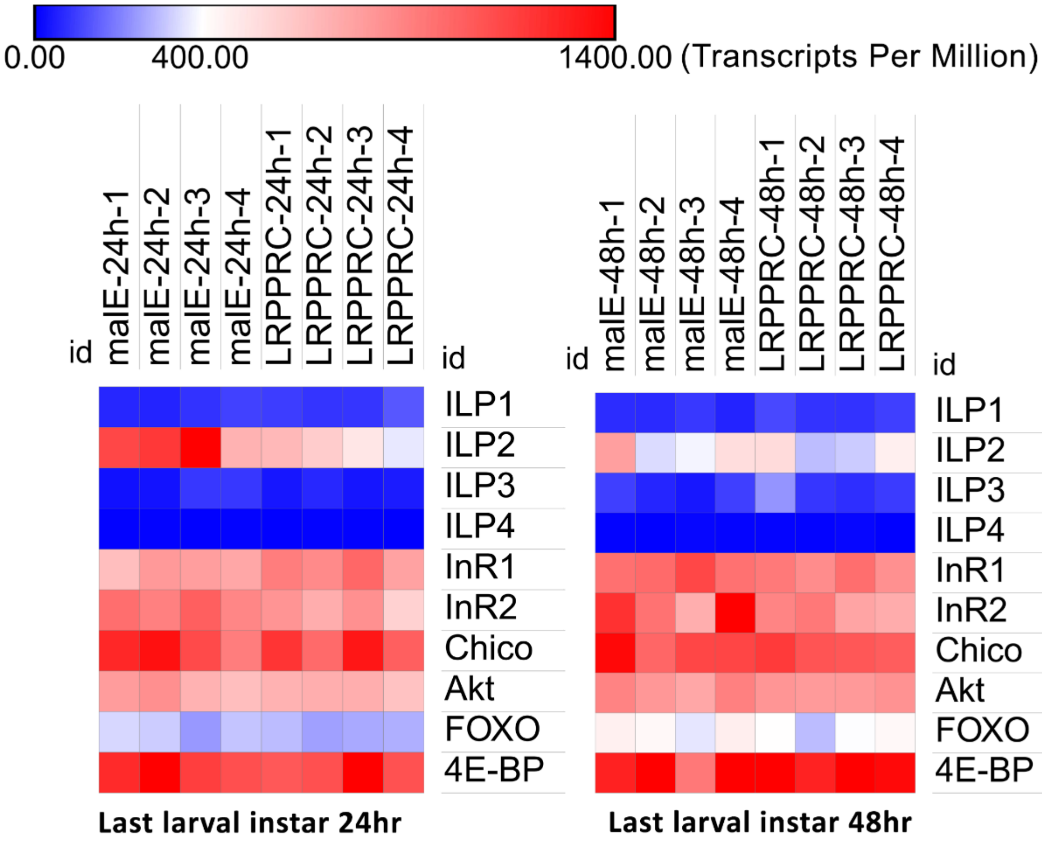


**Supplementary Figure 5.** Effect of mitochondrial dysfunction on IIS pathway. Heatmap showing the DEGs involved in IIS pathway after knockdown of *LRPPRC*. *dsmalE* or *dsTcLRPPRC* were injected to newly molted last instar larvae. 24h and 48h old larvae were sampled and processed for RNA-seq and DEG analysis. The heatmaps were prepared using Morpheus (https://software.broadinstitute.org/morpheus). The color code indicates the abundance of mRNA levels displayed by TPM (transcripts per million). The gene names are shown on the right side of the heatmap. The IDs of these genes are listed in Table S2. Four replicates were used for each treatment.

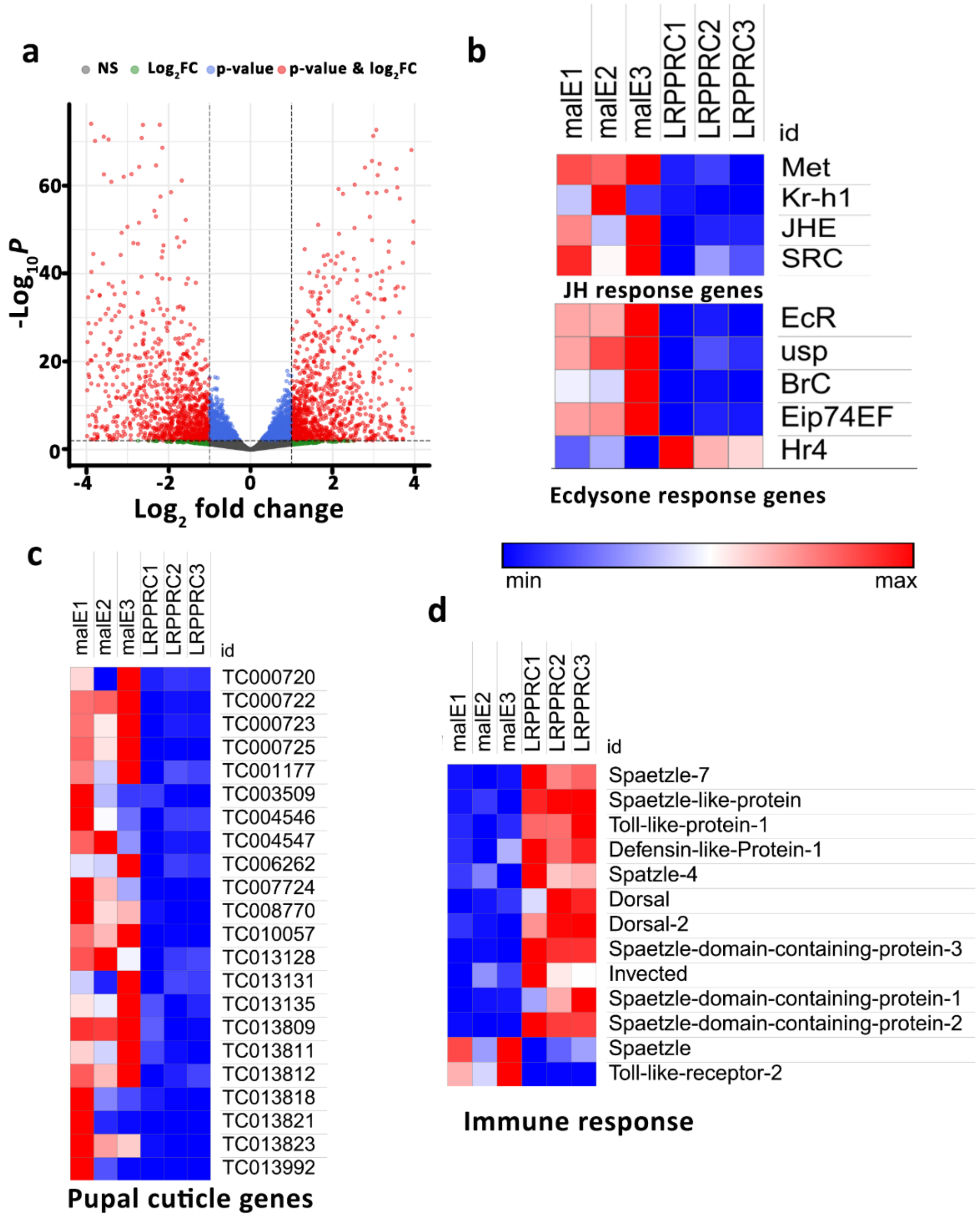


**Supplementary Figure 6. a.** Volcano plot showing differentially expressed genes in prepupal wing discs of larvae injected with *dsTcLRPPRC* or *dsmalE*. The red dots indicate a significant difference with over 2-fold change. **b.&c.** Heatmap showing insect hormone response and pupal cuticle genes are downregulated in prepupal wing discs upon knockdown of *TcLRPPRC*. **d.** Immune response genes are upregulated in prepupal wing discs upon knockdown of *TcLRPPRC*. The color code indicates the relative mRNA levels. The gene names are shown on the right side of the heatmap. Gene ID of these genes was listed in Table S2. Three replicates were used for each treatment and each replicate includes wing discs from 10 insects.

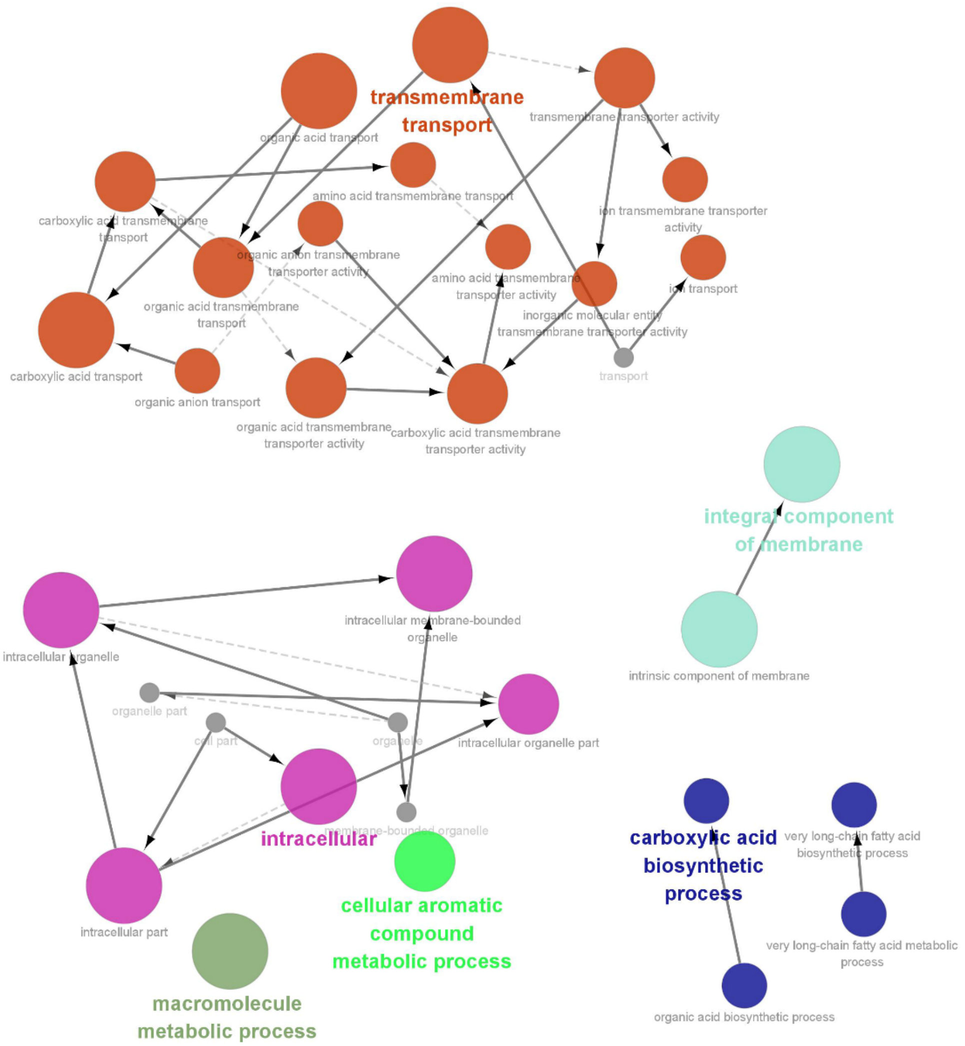


**Supplementary Figure 7.** Gene ontology (GO) enrichment analysis of significantly downregulated genes identified in RNA-seq of prepupal wing discs upon *TcLRPPRC* knockdown. Functional enriched GO network analysis was conducted by ClueGO. Each circle indicates a node, i.e., a specific GO term and color of the node represents a GO group.

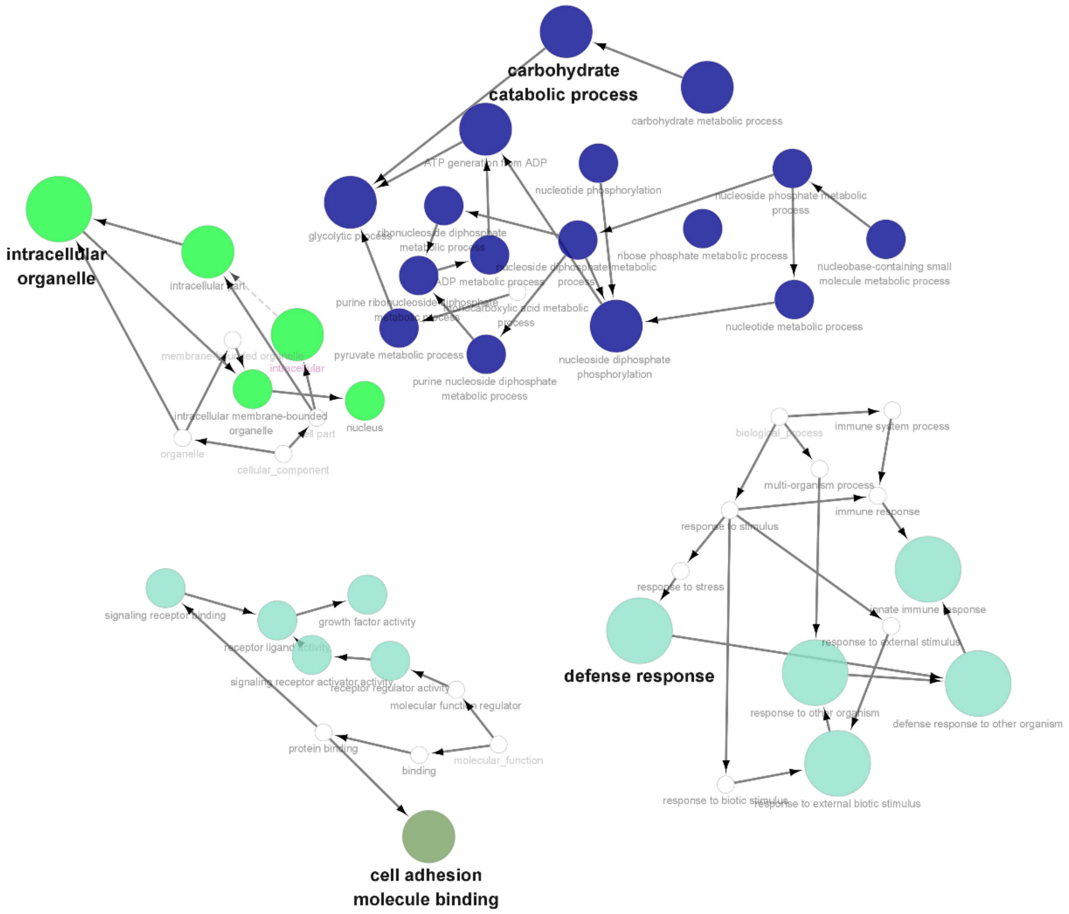


**Supplementary Figure 8.** Gene ontology (GO) enrichment analysis of significantly upregulated genes identified in RNA-seq of prepupal wing discs upon *TcLRPPRC* knockdown. Functional enriched GO network analysis was conducted by ClueGO. Each circle indicates a node, i.e., a specific GO term and color of the node represents a GO group.

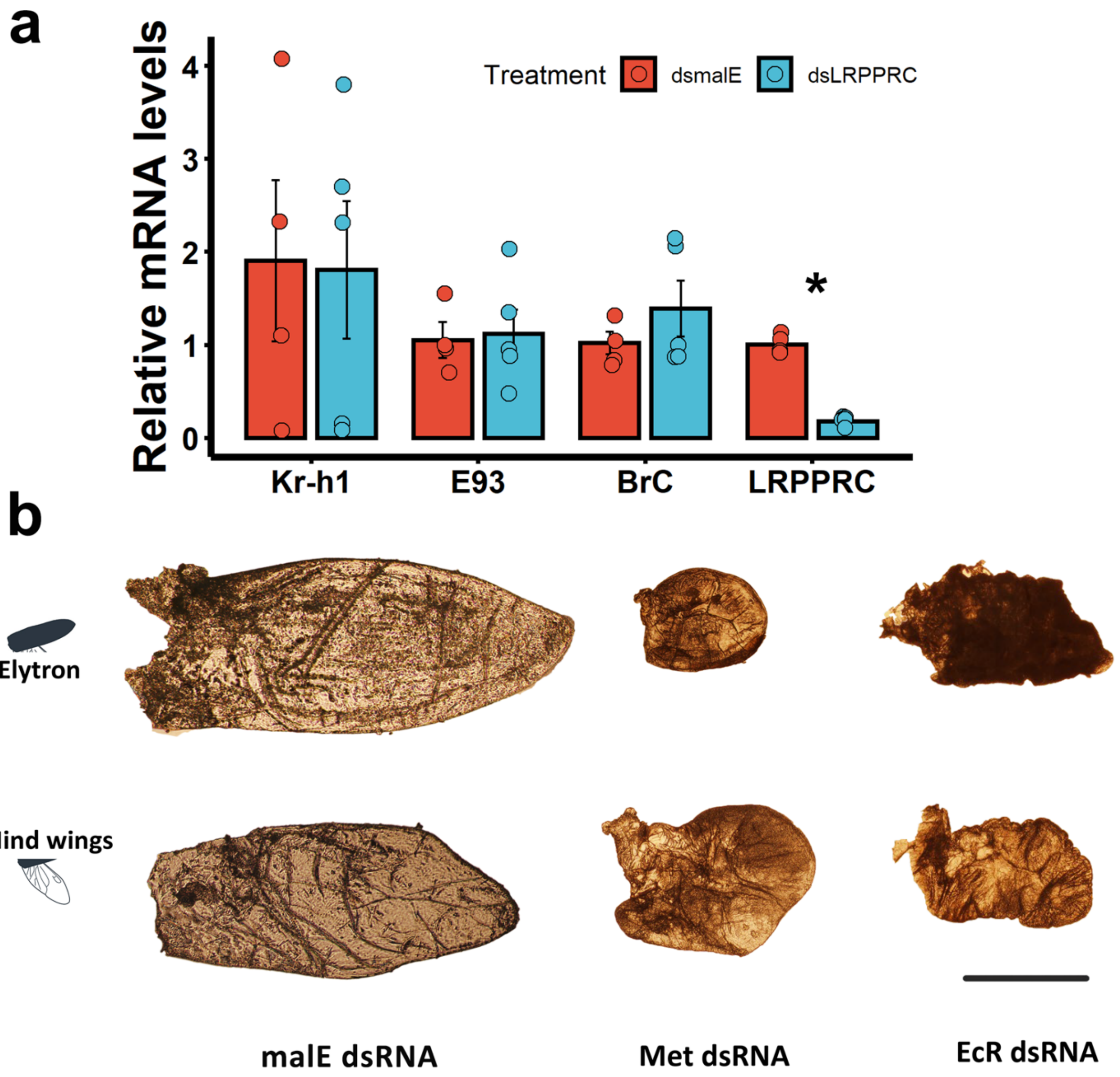


**Supplementary Figure 9. a.** Knockdown of *TcLRPPRC* in last instar larvae does not affect whole-body expression of genes involved in JH and Ecdysone action. Newly molted last instar larvae were injected with *malE* or *TcLRPPRC* dsRNA. Total RNA was extracted from larvae at 72 h after injection and used to quantify mRNA levels of *TcKr-h1*, *TcBrC*, *TcE93* and *TcLRPPRC*. The symbol * indicates significant difference between two treatments; $P < 0.05$. Means ± SE is shown. **b.** Pictures of elytron and hind wings of insects treated with dsRNAs of *malE*, *Met* (JH receptor) and *EcR* (ecdysone receptor). Insects were dissected and used to photograph when control insects turn into pupal stage. Scale bar = 200 μm.

**Supplementary Table 1.** Sequence of primers used in this study.

| Name | Seq |
|---|---|
| **dsRNA primers** | |
| Tc-TFAM-dsF | taatacgactcactatagggACAACTTAACCAAGCGTCGG |
| Tc-TFAM-dsR | taatacgactcactatagggGTCCGCGTATTCCTCCATCT |
| Tc-mtRNApolymerase-dsF | taatacgactcactatagggTGACTGAGACAAACGCCAAA |
| Tc-mtRNApolymerase-dsR | taatacgactcactatagggCGCCAGGTTGTTGTAGGTGT |
| Tc-mtTFB2-dsF | taatacgactcactatagggGCAGCAAGACAAAGTGAAGC |
| Tc-mtTFB2-dsR | taatacgactcactatagggATTTTTCAGGGATGTCGTGC |
| Tc-mtTFB1-dsF | taatacgactcactatagggGGCAGTATAACCCGGTCCAT |
| Tc-mtTFB1-dsR | taatacgactcactatagggGCAAAACCCTCCTCGAAATTA |
| Tc-LRPPRC-dsF | taatacgactcactatagggCAAAGTCAGACGCGCAATC |
| Tc-LRPPRC-dsR | taatacgactcactatagggTTGGCTTTGACCATTTGTTTT |
| Tc-LRPPRC-dsF2 | taatacgactcactatagggGAGTTTTTGGCCGACTTGG |
| Tc-LRPPRC-dsR2 | taatacgactcactatagggCACGATAAGCGCATTGAAGA |
| Tc-InR1-dsF | taatacgactcactatagggAGAGACGTCCATCTCTGTTGG |
| Tc-InR1-dsR | taatacgactcactatagggAAACCCCATGCTGAGATTTTT |
| Tc-InR2-dsF | taatacgactcactatagggCCGGATTGATAGCTGCATTA |
| Tc-InR2-dsR | taatacgactcactatagggGCCCGCAAGTAAAACACGTA |
| Tc-FOXO-dsF | taatacgactcactatagggCCGCCAAGAAGAACTCCAG |
| Tc-FOXO-dsR | taatacgactcactatagggCCCTTATCCTTGAAGTAGGGC |
| | |
| **Poly A length measurement** | |
| Linker-DNA | 5'-phospho-ATGTGAGATCATGCACAGTCATA-3'-NH2 |
| ANTI-LIGN | GACTGTGCATGATCTCAC |
| Tc-CYTB-PolyA-F | AATTGGAGCACGTCCAGTAG |
| Tc-COX1-PolyA-F | CTCGTCAATTGGGTCAATC |
| Tc-ND1-PolyA-F | ATGTGGAGTACGGAAGAGGA |
| Tc-ND2-PolyA-F | CTTGCTATCACTAGGTGGACTAC |
| Tc-ND3-PolyA-F | GAATGCGGATTTGACCCTAAAG |
| Tc-ND4-PolyA-F | GCGTCTTCATAGACGTAGTCTTTAT |
| Tc-ND5-PolyA-F | GCCTGTAATTTCTACTTTGGGTATT |
| | |
| **qPCR primers** | |
| Tc-LRPPRC-qRT-F | TGTACTCAGCCGGGATTTATG |
| Tc-LRPPRC-qRT-R | GCCTTCCAGTTCTTTCAGTTTG |
| Tc-mt-Polymerase-qRT-F | GGACAACGGCGGGTATTT |
| Tc-mt-Polymerase-qRT-R | CTGCTGAACCTCACTCTGATAC |
| Tc-TFAM-qRT-F | TCCGCCAAAGCACACTAAA |
| Tc-TFAM-qRT-R | GACTACAATCCGCCGAACTAAA |
| Tc-mtTFB1-qRT-F | TCTTGGGCATTTACGGCTATTC |
| Tc-mtTFB1-qRT-R | GCCGCTGCCATTACTACTTT |

| | |
|---|---|
| Tc-COX1-qRT-F | TGGGCCCACCACATATTTAC |
| Tc-COX1-qRT-R | TCCGGTTGGAACAGCAATAA |
| Tc-ND1-qRT-F | ATGTGGAGTACGGAAGAGGA |
| Tc-ND1-qRT-R | GCACCCAGAAATAATATGACACAC |
| Tc-CYTB-qRT-F | AATTGGAGCACGTCCAGTAG |
| Tc-CYTB-qRT-R | GGTGTATGTGAGTGGGTTGAT |
| Tc-ND4-qRT-F | GCGTCTTCATAGACGTAGTCTTTAT |
| Tc-ND4-qRT-R | TTATTGAAGGAGGAGCAGCAATA |
| Tc-ND5-qRT-F | GCCTGTAATTTCTACTTTGGGTATT |
| Tc-ND5-qRT-R | CTACCCAGAACCTCAGATCAAC |
| Tc-ND2-qRT-F | CTTGCTATCACTAGGTGGACTAC |
| Tc-ND2-qRT-R | CGAGGGCGATTATTGAAAGTTG |
| Tc-COX2-qRT-F | CCGCCTCCTAGATGTTGATAAT |
| Tc-COX2-qRT-R | CTCCGAGTGATGGGATTGTT |
| Tc-ND3-qRT-F | GAATGCGGATTTGACCCTAAAG |
| Tc-ND3-qRT-R | AGGAAGATGATGGCAATTAGGA |
| Tc-FOXO-qRT-F | TCGCCGATACCAGCAGTTGT |
| Tc-FOXO-qRT-R | TGCCCTGTTCGAGGTTTCCT |
| Tc-InR1-qRT-F | CCTGGATTCGTTCAACAGGT |
| Tc-InR1-qRT-R | GATCGAGTTCACGAAGCACA |

**Supplementary Table 2.** Gene IDs, names, and corresponded protein names of the genes studied in this study.

| Gene id | Protein Name |
|---|---|
| | **Wing** |
| TC032751 | nub,nubbin |
| TC014350 | vvl, ventral veins lacking |
| TC032664 | apB, apterous |
| TC032662 | apA, apterous |
| TC033006 | vg, vestigial |
| TC008466 | dpp, Decapentaplegic |
| TC008433 | ash, Achaete-scute-complex-protein |
| TC008629 | hth, homothorax |
| TC031040 | ara, araucan |
| TC011785 | fng, fringe |
| TC032269 | hh, hedgehog |
| TC013707 | Protein-Wnt6 |
| TC010155 | Protein-Wnt |
| TC009318 | Protein-Wnt5 |
| | **Ecdysone** |
| TC031968 | Eip74EF |
| TC034305 | EcR, ecdysone receptor |
| TC005474 | BrC, Broad-complex |
| TC014027 | usp, ultraspiracle |
| TC000543 | HR4, Hormone receptor 4 |
| | **JH** |
| TC012990 | kr-h1, Krueppel-homolog-1-like-Protein |
| TC003908 | Met, Methoprene-tolerant |
| TC013193 | JHE,Carboxylic-ester-hydrolase |
| TC014256 | SRC, homologous to the mammalian steroid receptor coactivator 1 |
| | **Pupal cuticles** |
| TC013821 | Pupal-cuticle-protein-Edg-84A-like-Protein |
| TC013992 | Pupal-cuticle-protein-Edg-84A-like-Protein |
| TC007724 | Pupal-cuticle-protein-Edg-84A-like-Protein |
| TC013823 | Pupal-cuticle-protein-Edg-84A-like-Protein |
| TC010057 | Pupal-cuticle-protein-Edg-84A-like-Protein |
| TC002434 | Cuticle-protein-19-like-Protein |
| TC000725 | Pupal-cuticle-protein-Edg-84A-like-Protein |
| TC008770 | Pupal-cuticle-protein-Edg-84A-like-Protein |
| TC003509 | Pupal-cuticle-protein-Edg-84A-like-Protein |
| TC013811 | Pupal-cuticle-protein-Edg-84A-like-Protein |

| | |
|---|---|
| TC000722 | Pupal-cuticle-protein-Edg-84A-like-Protein |
| TC000723 | Pupal-cuticle-protein-Edg-84A-like-Protein |
| TC004546 | Cuticle-protein-3-like-Protein |
| TC013809 | Pupal-cuticle-protein-Edg-84A-like-Protein |
| TC013818 | Pupal-cuticle-protein-Edg-84A-like-Protein |
| TC004547 | Endocuticle-structural-glycoprotein-SgAbd-2-like-Protein |
| TC013812 | Pupal-cuticle-protein-Edg-84A-like-Protein |
| TC006262 | Pupal-cuticle-protein-C1B-like-Protein |
| TC013131 | Pupal-cuticle-protein-like-Protein |
| TC000720 | Pupal-cuticle-protein-Edg-84A-like-Protein |
| TC013128 | Pupal-cuticle-protein-like-Protein |
| TC001177 | Larval/pupal-cuticle-protein-H1C-like-Protein |
| TC013135 | Pupal-cuticle-protein-Edg-78E-like-Protein |
| **Programmed cell death** | |
| TC034408 | Uncharacterized protein |
| TC031980 | XK-related protein |
| TC006266 | charybde-like Protein |
| TC008566 | Serine-protein kinase ATM-like Protein |
| TC011076 | Endonuclease G |
| TC012581 | Caspase Nc-like Protein |
| TC004531 | XK-related protein |
| TC000475 | Cell death activator CIDE-A-like Protein |
| TC013535 | XK-related protein |
| TC008059 | Protein tumorous imaginal discs, mitochondrial-like Protein |
| TC005774 | DnaJ homolog subfamily C member 28-like Protein |
| TC005886 | Protein charybde-like Protein |
| **Immune system** | |
| TC032149 | Spaetzle domain-containing protein 1 |
| TC033974 | Toll-like receptor 2 |
| TC032150 | Spaetzle domain-containing protein 2 |
| TC006726 | Spatzle 4 |
| TC001054 | Spaetzle-like protein |
| TC000520 | Spaetzle |
| TC001053 | Spatzle 7 |
| TC004439 | Toll-like protein 1 |
| TC004452 | Toll-like protein 2 |
| TC016368 | Invected |
| TC013304 | Spaetzle domain-containing protein 3 |
| TC007697 | Dorsal |
| TC008096 | Dorsal 2 |
| TC006250 | Defensin-like Protein 1 |

| | |
|---|---|
| TC012469 | Defensin-like Protein 2 |
| **IIS pathway** | |
| TC008479 | ILP1 |
| TC007035 | ILP2 |
| TC000934 | ILP3 |
| TC032112 | ILP4 |
| TC010784 | InR1 |
| TC007370 | InR2 |
| TC034013 | Chico |
| TC007749 | Akt |
| TC032156 | FOXO |
| TC006808 | 4E-BP |
