# Supplementary material for: Mitochondria dysfunction impairs Tribolium castaneum wing development during metamorphosis
Source: Commun Biol. 2022 Nov 15;5:1252. doi: 10.1038/s42003-022-04185-z (PMC9666433; doi:10.1038/s42003-022-04185-z)
Supplement: Supplementary file 2 — Description of Additional Supplementary Files [file 42003_2022_4185_MOESM2_ESM.pdf]

Description of Additional Supplementary Files

File name: Supplementary Data 1

Description: All the differentially expressed genes (DEGs) with p-value adjusted (padj) $\leq$ 0.05 at 24 h in dsTcLRPPRC treated larvae when compared to that in dsmalE treated control larvae.

File name: Supplementary Data 2

Description: All the differentially expressed genes (DEGs) with padj $\leq$ 0.05 at 48 h in dsTcLRPPRC treated larvae when compared to that in dsmalE treated control larvae.

File name: Supplementary Data 3

Description: All the differentially expressed genes (DEGs) with $\geq$2-fold change and padj $\leq$ 0.05 in wing discs dissected from dsTcLRPPRC treated insects when compared to that in control insects.

File name: Supplementary Data 4

Description: Source data behind the graphs in the main figures.
