# Supplementary material for: Mitochondria dysfunction impairs Tribolium castaneum wing development during metamorphosis
Source: Commun Biol. 2022 Nov 15;5:1252. doi: 10.1038/s42003-022-04185-z (PMC9666433; doi:10.1038/s42003-022-04185-z)
Supplement: Supplementary file 7 — Reporting Summary [file 42003_2022_4185_MOESM7_ESM.pdf]

nature portfolio

Corresponding author(s): Subba Reddy Palli
Last updated by author(s): Oct 27, 2022

Reporting Summary

Nature Portfolio wishes to improve the reproducibility of the work that we publish. This form provides structure for consistency and transparency in reporting. For further information on Nature Portfolio policies, see our Editorial Policies and the Editorial Policy Checklist.

Statistics

For all statistical analyses, confirm that the following items are present in the figure legend, table legend, main text, or Methods section.

n/a	Confirmed	
☐	☒	The exact sample size (*n*) for each experimental group/condition, given as a discrete number and unit of measurement
☐	☒	A statement on whether measurements were taken from distinct samples or whether the same sample was measured repeatedly
☐	☒	The statistical test(s) used AND whether they are one- or two-sided<br>*Only common tests should be described solely by name; describe more complex techniques in the Methods section.*
☒	☐	A description of all covariates tested
☐	☒	A description of any assumptions or corrections, such as tests of normality and adjustment for multiple comparisons
☐	☒	A full description of the statistical parameters including central tendency (e.g. means) or other basic estimates (e.g. regression coefficient) AND variation (e.g. standard deviation) or associated estimates of uncertainty (e.g. confidence intervals)
☐	☒	For null hypothesis testing, the test statistic (e.g. *F*, *t*, *r*) with confidence intervals, effect sizes, degrees of freedom and *P* value noted<br>*Give P values as exact values whenever suitable.*
☒	☐	For Bayesian analysis, information on the choice of priors and Markov chain Monte Carlo settings
☒	☐	For hierarchical and complex designs, identification of the appropriate level for tests and full reporting of outcomes
☒	☐	Estimates of effect sizes (e.g. Cohen's *d*, Pearson's *r*), indicating how they were calculated

Our web collection on statistics for biologists contains articles on many of the points above.

Software and code

Policy information about availability of computer code

Data collection	LAS X was used to analyze confocal data;<br>The StepOne™ Software was used for RT-qPCR data;<br>Typhoon 9500 (GE Healthcare Life Sciences) was used to scan phosphor image;<br>NIS-Elements was used for the measurement of wings and body size;<br>Statistics was conducted in R.
Data analysis	Codes of STAR, RSEM, DEseq2 in RNA-seq analyses can be found in their manual. The analyses were conducted in Lipscomb Compute Cluster at University of Kentucky;<br>ClueGo in Cytoscape was used for gene ontology biological process;<br>clusterProfiler package in R platform was used for Kyoto Encyclopedia of Genes and Genomes analyses.

For manuscripts utilizing custom algorithms or software that are central to the research but not yet described in published literature, software must be made available to editors and reviewers. We strongly encourage code deposition in a community repository (e.g. GitHub). See the Nature Portfolio guidelines for submitting code & software for further information.

Data

Policy information about availability of data

All manuscripts must include a data availability statement. This statement should provide the following information, where applicable:

- Accession codes, unique identifiers, or web links for publicly available datasets
- A description of any restrictions on data availability
- For clinical datasets or third party data, please ensure that the statement adheres to our policy

The transcriptomic raw data can be found under BioProject PRJNA732477 in NCBI.

Human research participants

Policy information about studies involving human research participants and Sex and Gender in Research.

Reporting on sex and gender	n/a
Population characteristics	n/a
Recruitment	n/a
Ethics oversight	n/a

Note that full information on the approval of the study protocol must also be provided in the manuscript.

Field-specific reporting

Please select the one below that is the best fit for your research. If you are not sure, read the appropriate sections before making your selection.

☒ Life sciences ☐ Behavioural & social sciences ☐ Ecological, evolutionary & environmental sciences

For a reference copy of the document with all sections, see nature.com/documents/nr-reporting-summary-flat.pdf

Life sciences study design

All studies must disclose on these points even when the disclosure is negative.

Sample size	At least three biological replicates were used for all the study. The statistical significance was tested for the results. The detailed sample size was described in the figure legends.
Data exclusions	There are no specific data exclusions for data analyses.
Replication	Most of findings from this study including phenotypes, gel pictures, RT-qPCRs in main text were all confirmed by another independent experiment.
Randomization	Insects were randomly taken from a pool of lab colony. When conducting insect microinjections, the sequence for both treatments and animals were also random.
Blinding	The investigators were not blinded to group allocations during data collection. Multiple treatments were used in the study. Treated insects need to be carefully traced to collect data, thus it is not possible to conduct bioassay without assigning groups.

Reporting for specific materials, systems and methods

We require information from authors about some types of materials, experimental systems and methods used in many studies. Here, indicate whether each material, system or method listed is relevant to your study. If you are not sure if a list item applies to your research, read the appropriate section before selecting a response.

Materials & experimental systems

n/a	Involved in the study	
☒	☐	Antibodies
☐	☒	Eukaryotic cell lines
☒	☐	Palaeontology and archaeology
☐	☒	Animals and other organisms
☒	☐	Clinical data
☒	☐	Dual use research of concern

Methods

n/a	Involved in the study	
☒	☐	ChIP-seq
☒	☐	Flow cytometry
☒	☐	MRI-based neuroimaging

Eukaryotic cell lines

Policy information about cell lines and Sex and Gender in Research

Cell line source(s)	TcA cell lines. This cell lines was developed from late pupae of the red flour beetle. https://doi.org/10.1038/srep06840
Authentication	The cells were authenticated by PCR amplification of select genes.
Mycoplasma contamination	Cells were not tested for mycoplasma contamination.
Commonly misidentified lines (See ICLAC register)	n/a

Animals and other research organisms

Policy information about studies involving animals; ARRIVE guidelines recommended for reporting animal research, and Sex and Gender in Research

Laboratory animals	The red flour beetle, Tribolium castaneum. Strains: GA-1 strain and pu-11 strain.
Wild animals	n/a
Reporting on sex	Findings apply to both male and female. A pool of mixed insects were used except for indication in the MS.
Field-collected samples	n/a
Ethics oversight	No ethics oversight was required by institution since no vertebrate animals were used in this study.

Note that full information on the approval of the study protocol must also be provided in the manuscript.
